# Predicting the Effects of Random Ocean Dynamic Processes on Underwater Acoustic Sensing and Communication

**DOI:** 10.1038/s41598-020-61043-w

**Published:** 2020-03-11

**Authors:** Byunggu Cho, Nicholas C. Makris

**Affiliations:** Center for Ocean Engineering, Massachusetts Institute of Technology, 77 Mass. Ave., Cambridge, MA 02139 USA

**Keywords:** Physical oceanography, Acoustics

## Abstract

Acoustics is the primary means of sensing and communication in the ocean for humans and many marine animals. Natural fluctuations in the ocean, however, degrade these abilities in ways that have been previously difficult to forecast. Here, we address this issue by predicting sensing and communication degradation in terms of acoustic attenuation, dispersion and temporal decorrelation at typical operational ranges and frequencies in continental-shelf environments. This is done with analytic expressions derived from first physical principles. The analytic expressions provide the statistics of the acoustic field after forward propagating through an ocean waveguide containing 3-D random inhomogeneities from the independent or combined effects of rough sea-surfaces, near-sea-surface air bubbles and internal waves. The formulation also includes Doppler effects caused by the inhomogeneities’ random horizontal motion, enabling modeling and prediction over a wide range of environments and frequencies. Theoretical predictions are confirmed with available acoustic measurements in several continental-shelf environments using standard oceanographic measurements for environmental support. We quantify how the acoustic signals decorrelate over timescales determined by the underlying temporal coherence of ocean dynamic processes. Surface gravity waves and near-sea-surface air bubbles decorrelate acoustic signals over seconds or less, whereas internal waves affect acoustic coherence at timescales of several to tens of minutes. Doppler spread caused by the inhomogeneities’ motion further reduces acoustic temporal coherence, and becomes important at the high frequencies necessary for communication and fine-scale sensing. We also show that surface gravity waves and bubbles in high sea states can cause increasingly significant attenuation as frequency increases. The typical durations of marine mammal vocalizations that carry over great distances are found to be consistent with the coherence timescales quantified here and so avoid random distortion of signal information even by incoherent reception.

## Introduction

Acoustics is the primary means of sensing and communication in the ocean for applications in such diverse areas as ocean resource management, marine ecology, climatology, oceanography and national defense^1–9^. This is due to the severe attenuation of electromagnetic waves in water^3^. Current ocean sensing limitations make marine resource management challenging. Without significant improvements in ocean sensing it will be difficult to address the unprecedented decline in many oceanic species recently described and predicted by the United Nations^10^. Many marine animals also use acoustics to communicate, navigate, and locate food^11–13^. Natural fluctuations and resulting inhomogeneities in the ocean such as surface waves, internal waves and bubbles, however, can significantly degrade acoustic sensing and communication abilities^14–18^ by introducing attenuation, dispersion and coherence losses that limit operational ranges, time windows and frequency bands. Acoustic temporal coherence loss, for example, shortens the time window within which standard coherent processing can be conducted and so bounds sensing and communication ranges. Acoustic time scales when shorter, however, can be advantageous in the context of stationary averaging for signal variance reduction since they increase the number of independent samples within a fixed measurement time window^4,19^, making their accurate prediction important in determining the possible resolution of ocean parameters to be sensed.

In the present work, we predict attenuation, dispersion and temporal coherence for general ocean acoustic sensing and communication applications in continental-shelf environments via an analytic full-field formulation derived from first principles. The analytic approach provided here is suitable for a wide range of frequencies and ocean environments from continental shelves to deep basins at various sea states, where other approximate approaches^16,20,21^ are not applicable. The analytic expressions include full 3-D multiple forward scattering effects from potentially rough sea-surfaces at high sea states with the presence of near-sea-surface air bubbles. Scattering from large-amplitude surface gravity waves is modeled using the small slope approximation^22^ and that from near-surface bubble clouds includes the effects of resonance^23^. The formulation also includes Doppler effects caused by the inhomogeneities’ random horizontal motion. Unlike forward propagation in free space, we show that the inhomogeneities’ motion in a waveguide causes Doppler spread in the forward field due to off-diagonal acoustic mode coupling and reduces acoustic temporal coherence. For internal waves, the accumulated scattering effects from these Rayleigh-Born inhomogeneities are modeled using existing analytic expressions^24,25^. This analytic model has been used to explain attenuation and temporal coherence reduction in a number of ocean acoustic measurements in abyssal-plain and continental-shelf environments containing random internal wave fields^24–26^.

We quantify the combined effects of surface gravity waves, near-sea-surface air bubbles and internal waves because of their relevance to typical acoustic sensing^27,28^ and communication^29,30^ systems, and find them to be primary causes of acoustic sensing and communication degradations in the ocean. We show that surface gravity waves can cause significant attenuation and temporal coherence loss by randomizing the ocean’s free upper surface. Air bubbles near the sea surface are damped-forced oscillators that degrade the acoustic signal through scattering, especially as the acoustic transmission frequency approaches the resonance scattering frequency of these bubbles. As sea state increases, we find that scattering from sea surface and bubbles increasingly limits underwater acoustic sensing and communication capabilities. Internal waves randomize the ocean medium compressibility and density and can cause an accumulated effect on acoustic signals propagating over long ranges through the ocean^16,17,31,32^. The independent or combined effects of random ocean dynamic processes on acoustic field statistics are quantified using standard oceanographic measurements, such as sea state, particle velocity at the sea surface, bubble number density and internal wave energy. Predictions are compared with available acoustic measurements in continental-shelf environments in the Gulf of Mexico, Barents sea and Bristol channel. We also show medium motion from surface gravity wave’s orbital particle velocity and Stoke’s drift have a negligible effect on acoustic temporal coherence and attenuation.

Our analysis follows Rayleigh’s classic free space approach^20^, employing differential slabs of scatterers, which has been generalized to determine the first and second moments of the acoustic field after propagating through 3-D inhomogeneities in a waveguide^25,33^. Inhomogeneities of arbitrary size or contrast in compressibility and density are allowed because the generalization is based on a full-field waveguide scattering approach^34,35^. This generalized analytic approach (i) provides a physical understanding of the effects of ocean dynamic processes on acoustic propagation (ii) requires only a few typical oceanographic measurements for predictions and (iii) can be applied to a broader range of environments and applications than previous approaches. Many free space atmospheric^20,36–40^ and related ray approximations^16,17^ do not include ocean boundaries which are the dominant cause of waveguide effects in continental-shelf environments and neglect important diffractive field components. The effects of 3-D inhomogeneities with scales smaller than the acoustic Fresnel width have been shown to lead to multiple out-of-plane scattering effects that significantly degrade sensing and communication abilities^24–26,33^, which are neglected in 2-D approaches. Perturbation theory based models^21,41–45^ either require surface roughness and slope to be small or variations in medium sound speed and density to be small, which is often not the case in many sensing and communication scenarios, especially in high sea states.

## Results

### Analytic expressions for the mean and temporal correlation of the acoustic forward field in a waveguide containing moving 3-D random inhomogeneities

In this section, we provide the physics and fundamental assumptions behind analytic expressions for the mean and temporal correlation of the forward field propagated through moving random inhomogeneities in a 3-D waveguide. These analytic expressions are advantageous because they (i) isolate physical mechanisms and enable clear interpretations, (ii) yield direct statistics of the field and (iii) provide accurate predictions for a broad range of applications in the ocean.

Following previous work on forward propagation in a waveguide with random inhomogeneities^33^, the mean forward field in an ocean waveguide can be analytically marched through successive differential range slabs of moving inhomogeneities (Supplementary Fig. S1) to include multiple forward scattering. This is possible when (i) the field scattered from the inhomogeneities within any single differential range slab is small compared to the incident field; (ii) the thickness of any differential range slab of moving inhomogeneities is sufficiently small for single scatter approximation to be valid within it and sufficiently large for modal decoupling to occur in the mean forward field; (iii) the inhomogeneities move much slower than the sound speed over the time period the acoustic wave passes through it; and (iv) the medium’s 3-D inhomogeneities follow a stationary random process over the acoustic measurement time and within the horizontal area defined by the slab thickness and the range-dependent Fresnel width, but need not follow a stationary random process in the vertical or across successive range slabs. Forward scattering is dominated by contributions from within the cross-range Fresnel width between the source and receiver, as determined analytically by stationary phase analysis similar to the method used by Rayleigh^20^ and van de Hulst^36^ in free space optics. The resulting mean total forward field at a point receiver ${\bf{r}}$, $\langle {\Phi }_{T}\left({\bf{r}}| {{\bf{r}}}_{{\bf{0}}}\right)\rangle$, takes the form of a product of the incident field and an exponential factor with a complex phase that accumulates horizontal wavenumber change due to scattering from source to receiver as

1 $$\left\langle {\Phi }_{T}\left({\bf{r}}| {{\bf{r}}}_{{\bf{0}}}\right)\right\rangle =\sum _{n}{\Phi }_{i}^{(n)}\left({\bf{r}}| {{\bf{r}}}_{{\bf{0}}}\right)\,\exp \,\left[i{\int }_{0}^{\rho }{\nu }_{n}({\rho }_{s})d{\rho }_{s}\right]$$

where ${{\bf{r}}}_{{\bf{0}}}$ is the position of a monochromatic point source, $\rho$ is the horizontal range from the source to the receiver and ${\Phi }_{i}^{(n)}({\bf{r}}| {{\bf{r}}}_{{\bf{0}}})$ is the ${n}^{\mathrm{th}}$ modal component of the incident field (Supplementary Eq. (S10)). The horizontal complex wavenumber change, ${\nu }_{n}({\rho }_{s})$, accounts for dispersion and attenuation caused by scattering through the inhomogeneities at slab range ${\rho }_{s}$ (Supplementary Fig. S1), where the analytic expressions are shown in Eqs. (4), (5) and (6).

A similar marching procedure is used to derive temporal correlation of the acoustic power (Supplementary Eq. (S22)) and forward acoustic field (Eq. (2)). The incremental change in temporal correlation of the acoustic power due to a single range slab of moving inhomogeneities can be expressed in terms of the depth integral of the temporal correlation of the scattered field, as well as cross terms between the incident and scattered fields. This change is determined over the same Fresnel width that defines the mean forward field for consistency and can be expressed as the product of the incident power, slab thickness, and the difference between modal coefficients of field-temporal-covariance and attenuation (Supplementary Eq. (S21)). Temporal correlation of the acoustic power at the receiver range is then obtained by marching the incremental change in temporal correlation of the power from source to receiver through direct integration. This temporal correlation of the power is expressed as a product of the incident power and an exponential factor that involves range integration of the difference between modal coefficients of field-temporal-covariance and attenuation from source to receiver (Supplementary Eq. (S22)). When the acoustic modes are statistically uncorrelated, the temporal correlation of the acoustic forward field at a point receiver ${\bf{r}}$ at an acoustic time lag of $\tau ={{\mathfrak{t}}}_{1}-{{\mathfrak{t}}}_{2}$ is then obtained as

2 $$\begin{array}{rcl}{\mathrm{Corr}}_{{\Phi }_{T},{\Phi }_{T}}({\bf{r}},\tau | {{\bf{r}}}_{{\bf{0}}}) & = & \langle {\Phi }_{T}({\bf{r}},{{\rm{t}}}_{1}| {{\bf{r}}}_{{\bf{0}}}){\Phi }_{T}^{* }({\bf{r}},{{\rm{t}}}_{2}| {{\bf{r}}}_{{\bf{0}}})\rangle \\  & = & {| \langle {\Phi }_{T}({\bf{r}}| {{\bf{r}}}_{{\bf{0}}})\rangle | }^{2}+\sum _{n}{| {\Phi }_{i}^{(n)}({\bf{r}}| {{\bf{r}}}_{{\bf{0}}})| }^{2}\\  &  & \times {\rm{\exp }}\left[-2{\int }_{0}^{\rho }\Im \{{\nu }_{n}({\rho }_{s})\}d{\rho }_{s}\right]\\  &  & \times \left({\rm{\exp }}\left[{\int }_{0}^{\rho }{\mu }_{n}({\rho }_{s},\tau )d{\rho }_{s}\right]-1\right),\end{array}$$

where ${\mu }_{n}({\rho }_{s},\tau )$ is the modal field-temporal-covariance coefficient (Eqs. (7) and (8)), which quantifies decorrelation of the forward field over time. The acoustic temporal coherence function is defined as the normalized acoustic temporal correlation as

3 $$\varrho \left({\bf{r}},\tau | {{\bf{r}}}_{{\bf{0}}}\right)=\frac{\langle {\Phi }_{T}\left({\bf{r}},{{\mathfrak{t}}}_{1}| {{\bf{r}}}_{{\bf{0}}}\right){\Phi }_{T}^{* }\left({\bf{r}},{{\mathfrak{t}}}_{2}| {{\bf{r}}}_{{\bf{0}}}\right)\rangle }{\langle {\Phi }_{T}\left({\bf{r}},{{\mathfrak{t}}}_{1}| {{\bf{r}}}_{{\bf{0}}}\right){\Phi }_{T}^{* }\left({\bf{r}},{{\mathfrak{t}}}_{1}| {{\bf{r}}}_{{\bf{0}}}\right)\rangle },$$

where $\langle {\Phi }_{T}\left({\bf{r}},{{\mathfrak{t}}}_{1}| {{\bf{r}}}_{{\bf{0}}}\right){\Phi }_{T}^{* }\left({\bf{r}},{{\mathfrak{t}}}_{2}| {{\bf{r}}}_{{\bf{0}}}\right)\rangle$ is the acoustic temporal correlation defined in Eq. (2). The 0.8-crossing and e-folding acoustic coherence timescales, ${\tau }_{0.8}$ and ${\tau }_{e}$, are defined as the acoustic time delays at which $\varrho \left({\bf{r}},\tau | {{\bf{r}}}_{{\bf{0}}}\right)$ respectively crosses and falls below 0.8 and 1/$e$.

The real part of the modal horizontal wavenumber change leads to dispersion and is expressed as

4 $$\Re \{{\nu }_{n}\left({\rho }_{s}\right)\}=\Re \left\{\frac{1}{{\xi }_{n}}{\int }_{0}^{H}d{z}_{t}^{0}\left\langle {F}_{{z}_{t}^{0}}(n,n;0,0)\right\rangle \right\}.$$

The imaginary part of this modal horizontal wavenumber change, which causes attenuation, can be expressed either as

5 $$\Im \{{\nu }_{n}^{{\rm{2}}{\rm{-}}{\rm{D}}}({\rho }_{s})\}=\frac{1}{2}{\mu }_{n}^{2-D}({\rho }_{s},\tau =0)$$

or

6 $$\begin{array}{rcl}\Im \{{\nu }_{n}^{3-{\rm{D}}}({\rho }_{s})\} & = & \frac{1}{4\pi }\mathop{\sum }\limits_{m=1}^{\infty }\Re \left\{\frac{{\xi }_{m}^{* }}{| {\xi }_{m}| \Re \{{\xi }_{n}\}}{\int }_{0}^{H}d{z}_{t}^{0}{\int }_{0}^{H}d{z}_{{t}^{{\prime} }}^{0}{A}_{c}({\rho }_{s},{z}_{t}^{0},{z}_{{t}^{{\prime} }}^{0})\right.\\  &  & \left.\times {\int }_{0}^{2\pi }\langle {F}_{{z}_{t}^{0}}(m,n,\beta ,0){F}_{{z}_{{t}^{{\prime} }}^{0}}^{* }(m,n,\beta ,0)\rangle d\beta \right\}\end{array}$$

depending on the relative size of the cross-range coherence length of the random inhomogeneities with respect to the local Fresnel width. At slab ranges close to the source or receiver where the cross-range coherence length of the inhomogeneities exceeds the local Fresnel width, the inhomogeneities are fully correlated and 2-D forward scattering occurs. At slab ranges further away from both source and receiver, the local Fresnel width can exceed the cross-range coherence length of the inhomogeneities and 3-D scattering occurs^33^. Assuming no power loss in the forward direction where 2-D scattering occurs^25^, the modal attenuation factor, $\Im \{{\nu }_{n}^{2-{\rm{D}}}({\rho }_{s})\}$, is expressed in Eq. (5), where ${\mu }_{n}^{2-{\rm{D}}}({\rho }_{s},\tau =0)$ is defined in Eq. (7). At slab ranges where 3-D scattering occurs, the modal attenuation factor is expressed in Eq. (6), where the waveguide extinction theorem^46^ is used. When 3-D scattering occurs, out-of-plane scattering becomes important and leads to power loss in the forward direction. The incident and outgoing modal horizontal wavenumber components are respectively ${\xi }_{n}$ and ${\xi }_{m}$, and ${A}_{c}\left({\rho }_{s},{z}_{t}^{0},{z}_{{t}^{{\prime} }}^{0}\right)$ is the horizontal coherence area of the inhomogeneities at depths ${z}_{t}^{0}$ and ${z}_{{t}^{{\prime} }}^{0}$. Coupling between the ${n}^{\mathrm{th}}$ incident and ${m}^{\mathrm{th}}$ outgoing modal plane wave components due to scattering from an inhomogeneity at depth ${z}_{t}^{0}$ is represented by ${F}_{{z}_{t}^{0}}(m,n;\beta ,{\beta }_{i})$ (Supplementary Eq. (S12)), where ${\beta }_{i}$ and $\beta$ are the respective horizontal azimuths of source-to-inhomogeneity and inhomogeneity-to-receiver with respect to the forward direction (Supplementary Fig. S1).

The modal field-temporal-covariance coefficient, ${\mu }_{n}({\rho }_{s},\tau )$, represents the energy transfer from the mean field to the covariance field caused by the inhomogeneities’ intrinsic temporal decorrelation and Doppler spread due to their motion. This ${\mu }_{n}({\rho }_{s},\tau )$ is expressed as

7 $$\begin{array}{rcl}{\mu }_{n}^{2-{\rm{D}}}({\rho }_{s},\tau ) & = & \sum _{m}\frac{1}{\left|{\xi }_{m}\right|}{\left(1+\frac{{\bar{u}}}{{C}_{m}^{g}}\right)}^{2}\frac{1}{{\xi }_{m}}{\int }_{0}^{H}d{z}_{t}^{0}\\  &  & \times {\int }_{0}^{H}d{z}_{{t}^{{\prime} }}^{0}\frac{4{\pi }^{2}{l}_{x}\left({\rho }_{s},{z}_{t}^{0},{z}_{{t}^{{\prime} }}^{0}\right)}{k({z}_{t}^{0})k\left({z}_{{t}^{{\prime} }}^{0}\right)d({z}_{t}^{0})d\left({z}_{{t}^{{\prime} }}^{0}\right)}\\  &  & \times {e}^{i\Re \{{\xi }_{n}-{\xi }_{m}\}{\bar{u}}\tau }{C}_{s,s}\left({\rho }_{s},{z}_{t}^{0},{z}_{{t}^{{\prime} }}^{0},m,n,\tau \right)\end{array}$$

at slab ranges where 2-D scattering occurs, and

8 $$\begin{array}{rcl}{\mu }_{n}^{3-{\rm{D}}}({\rho }_{s},\tau ) & = & \sum _{m}\frac{1}{\left|\,{\xi }_{m}\right|}{\left(1+\frac{{\bar{u}}}{{C}_{m}^{g}}\right)}^{2}\sqrt{\frac{\rho }{2\pi \Re \{{\xi }_{m}\}{\rho }_{s}(\rho -{\rho }_{s})}}\\  &  & \times {\int }_{0}^{H}d{z}_{t}^{0}{\int }_{0}^{H}d{z}_{{t}^{{\prime} }}^{0}\frac{4{\pi }^{2}{A}_{c}\left({\rho }_{s},{z}_{t}^{0},{z}_{{t}^{{\prime} }}^{0}\right)}{k({z}_{t}^{0})k\left({z}_{{t}^{{\prime} }}^{0}\right)d({z}_{t}^{0})d\left({z}_{{t}^{{\prime} }}^{0}\right)}\\  &  & \times {e}^{i\Re \{{\xi }_{n}-{\xi }_{m}\}{\bar{u}}\tau }{C}_{s,s}\left({\rho }_{s},{z}_{t}^{0},{z}_{{t}^{{\prime} }}^{0},m,n,\tau \right)\end{array}$$

at slab ranges where 3-D scattering occurs, where ${l}_{x}\left({\rho }_{s},{z}_{t}^{0},{z}_{{t}^{{\prime} }}^{0}\right)$ is the coherence length of the inhomogeneities at depths ${z}_{t}^{0}$ and ${z}_{{t}^{{\prime} }}^{0}$ in range direction, ${\bar{u}}$ is the inhomogeneities’ mean forward direction velocity, $k({z}_{t}^{0})$ is the acoustic wavenumber and $d({z}_{t}^{0})$ is the medium density. Effects of the inhomogeneities’ temporal decorrelation on acoustic temporal coherence are quantified by ${C}_{s,s}\left({\rho }_{s},{z}_{t}^{0},{z}_{{t}^{{\prime} }}^{0},m,n,\tau \right)$, where the definition follows Eq. (72) of ref. ^33^, except the covariance of the scatter function densities, ${s}_{{z}_{t}^{0}}$ and ${s}_{{z}_{{t}^{{\prime} }}^{0}}$, at zero time lag is replaced by the temporal covariance of the scatter function densities with an acoustic time lag of $\tau$. This term expresses coupling between the ${n}^{\mathrm{th}}$ incident and ${m}^{\mathrm{th}}$ outgoing modal plane wave components due to scattering from temporally decorrelating inhomogeneities at depths ${z}_{t}^{0}$ and ${z}_{{t}^{{\prime} }}^{0}$. Temporal coherence of these inhomogeneities are determined, for example, by changes in surface gravity waveheights, bubble size and density variations or internal wave density and compressibility fluctuations. Eqs. (7) and (8) are different from Eqs. (5) and (8) of ref. ^26^ by an amplification factor, ${(1+{\bar{u}}/{C}_{m}^{g})}^{2}$, and a Doppler spread term, ${e}^{i\Re \{{\xi }_{n}-{\xi }_{m}\}{\bar{u}}\tau }$. These analytic expressions for ${\mu }_{n}({\rho }_{s},\tau )$ show that acoustic temporal decorrelation can occur by (i) the intrinsic temporal decorrelation of the inhomogeneities through the term ${C}_{s,s}\left({\rho }_{s},{z}_{t}^{0},{z}_{{t}^{{\prime} }}^{0},m,n,\tau \right)$ and (ii) Doppler spread caused by the translational motion of the inhomogeneities through ${e}^{i\Re \{{\xi }_{n}-{\xi }_{m}\}{\bar{u}}\tau }$. Effects of both mechanisms accumulate through multiple forward scattering as shown by the integration from source to receiver in Eq. (2). The Doppler spread term leads to spectral broadening of the acoustic temporal correlation, where the spectral width is determined by the incident acoustic frequency, directions of the incident and outgoing modal plane wave components and the modal Mach number of the inhomogeneities’ mean forward direction velocity. This frequency spreading can be simply shown by linearizing the Doppler spread term, ${e}^{i\Re \{{\xi }_{n}-{\xi }_{m}\}{\bar{u}}\tau }\approx 1+i\Re \{{\xi }_{n}-{\xi }_{m}\}{\bar{u}}\tau$, and substituting the linearized expressions for ${\mu }_{n}({\rho }_{s},\tau )$ into Eq. (2). As an acoustic signal travels to greater ranges and Doppler spread accumulates, this can lead to significant acoustic temporal coherence reduction especially at high frequencies or Mach numbers. For inhomogeneities with zero forward direction velocity, the Doppler spread term and the amplification factor vanishes, and so the modal field-temporal-covariance coefficient reduces to Eqs. (5) and (8) of ref. ^26^.

The analytic expressions for ${\nu }_{n}({\rho }_{s})$ (Eqs. (4), (5) and (6)) and ${\mu }_{n}({\rho }_{s},\tau )$ (Eqs. (7) and (8)) require the first two statistical moments of the inhomogeneities’ scatter function densities. For surface gravity waves, temporal covariance of the scatter function density is calculated using small slope approximation^22^ (Supplementary Section S2), where the required spatial and temporal covariance of the surface waves are calculated using an isotropic Pierson-Moskowitz spectrum^47^. The mean scatter function density of surface gravity waves is zero since the mean surface waveheight is zero. For internal waves, their scatter function density is modeled using Rayleigh-Born approximation^25,26^ and the required spatial and temporal covariance of their waveheights are calculated using a Garret-Munk internal wave spectrum calibrated for shallow environments^48^. The scatter function density of near-sea-surface air bubble clouds is modeled as a damped-forced oscillator, where the measured bubble number density spectrum and spatial scales of the bubble clouds are used for the calculation (Supplementary Section S3).

### Effects of traveling surface gravity waves, bubbles and internal waves on acoustic temporal coherence

**Figure 1:**
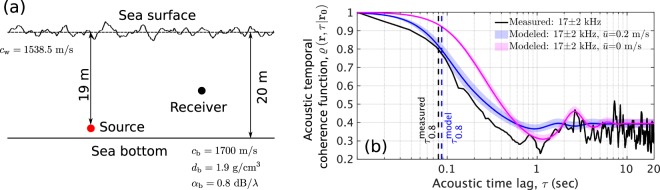
Acoustic temporal coherence loss caused by traveling surface gravity waves in an isovelocity continental-shelf environment. Both effects of underlying temporal decorrelation of surface waveheights and Doppler spread caused by their horizontal motion are shown. (**a**) A Gulf of Mexico continental-shelf environment with traveling surface gravity waves at a wind speed of 5 m/s (significant waveheight ${H}_{1/3}=0.56$ m and WMO sea state 3, Supplementary Table S1). A point source radiating at $17\pm 2$ kHz, 2.3 km from a point receiver at 18 m depth is used to compare predictions with measurements. (**b**) Comparison between predicted and measured acoustic temporal coherence functions, $\varrho ({\bf{r}},\tau | {{\bf{r}}}_{{\bf{0}}})$ (Eq. (3)), in the environment described in (**a**). The black solid line denotes the measured $\varrho \left({\bf{r}},\tau | {{\bf{r}}}_{{\bf{0}}}\right)$^28^. The blue shaded patch shows the modeled $\varrho \left({\bf{r}},\tau | {{\bf{r}}}_{{\bf{0}}}\right)$ including both effects of intrinsic temporal decorrelation of the surface waveheights and Doppler spread caused by their horizontal motion with mean forward velocity ${\bar{u}}=0.2$ m/s. The lower and upper limits of this shaded patch are respectively the predicted $\varrho ({\bf{r}},\tau | {{\bf{r}}}_{{\bf{0}}})$ at 19 kHz and 15 kHz, and the blue solid line is the modeled $\varrho \left({\bf{r}},\tau | {{\bf{r}}}_{{\bf{0}}}\right)$ at 17 kHz. The measured 0.8-crossing coherence timescale, ${\tau }_{0.8}=0.08$ seconds^28^, is consistent with the modeled ${\tau }_{0.8}=0.087\pm 0.013$ seconds. At longer time lags (>5 seconds), the measured $\varrho \left({\bf{r}},\tau | {{\bf{r}}}_{{\bf{0}}}\right)=0.35\pm 0.05$ is consistent with the predicted $\varrho \left({\bf{r}},\tau | {{\bf{r}}}_{{\bf{0}}}\right)=0.385\pm 0.025$. The red solid line and shaded patch are same as the blue solid line and shaded patch, but does not include Doppler effects.

Here, we quantify how acoustic temporal coherence is affected by processes that make the ocean an inhomogeneous acoustic medium. We begin by analyzing acoustic propagation in a typical isovelocity continental-shelf environment in the Gulf of Mexico at a wind speed of 5 m/s (significant waveheight ${H}_{1/3}=0.56$ m, WMO sea state 3) where multiple forward scattering from traveling surface gravity waves lead to acoustic temporal coherence loss (Fig. 1(a)). At this moderate sea state, the scattering effects of rough surface waves are expected to be notable only at high frequencies (>10 kHz). Our predictions (Eq. (3)) are consistent across the available acoustic temporal coherence data^28^, as can be seen in Fig. 1(b). Acoustic coherence at long time lags (>5 seconds) is found to be dominated by the correlation function of surface gravity waveheights via ${C}_{s,s}\left({\rho }_{s},{z}_{t}^{0},{z}_{{t}^{{\prime} }}^{0},m,n,\tau \right)$ in Eqs. (7) and (8). Acoustic coherence at short time lags (<1 second), on the other hand, is found to be dominated by Doppler spread through ${e}^{i\Re \{{\xi }_{n}-{\xi }_{m}\}{\bar{u}}\tau }$ in Eqs. (7) and (8). Doppler spread is proportional to acoustic frequency and becomes notable at frequencies of roughly 10 kHz and above (Fig. 1(b), Supplementary Section S5). The acoustic signal decorrelates more rapidly than the surface waveheights because of the accumulated effect of multiple forward scattering through many surface waves, as shown in Eq. (2). Ocean medium motion below the sea surface caused by surface gravity waves, wind-induced drift and currents are shown to have a negligible effect on acoustic temporal coherence, including Doppler spread, and attenuation in Supplementary Section S8.

**Figure 2:**
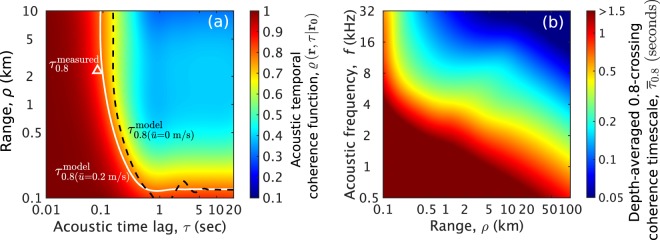
Prediction of acoustic temporal coherence loss caused by traveling surface gravity waves in an isovelocity continental-shelf environment (Fig. 1(a)) at general ranges and frequencies for ocean sensing and communication. A wind speed of 5 m/s (significant waveheight ${H}_{1/3}=0.56$ m and WMO sea state 3) is used. Both effects of surface waveheight decorrelation and Doppler spread from horizontal surface wave motion accumulate with increasing range and reduce acoustic coherence by more than 20% within seconds. Doppler effects become important at high frequencies for underwater communication and fine-scale sensing. (**a**) Acoustic temporal coherence function, $\varrho \left({\bf{r}},\tau | {{\bf{r}}}_{{\bf{0}}}\right)$ (Eq. (3)), predicted as a function of range and acoustic time lag at 17 kHz and 18 m receiver depth. The horizontal mean particle velocity at the sea surface in the forward direction ${\bar{u}}=0.2$ m/s. The white solid line denotes the modeled 0.8-crossing coherence timescale (${\tau }_{0.8}$). Similarly, the black dashed line shows the modeled ${\tau }_{0.8}$, but when Doppler effects are not included, i.e. ${\bar{u}}=0$. The white open triangle indicates the measured ${\tau }_{0.8}$^28^. (**b**) Depth-averaged 0.8-crossing coherence timescale (${\bar{\tau }}_{0.8}$) predicted as a function of range and acoustic frequency in the same environment. This timescale shortens with increasing frequency and range, where ${\bar{\tau }}_{0.8} < 1.5$ seconds is only shown. The region where ${\bar{\tau }}_{0.8} > 1.5$ seconds denotes frequencies and ranges where $\varrho \left({\bf{r}},\tau | {{\bf{r}}}_{{\bf{0}}}\right)$ does not fall below 0.8. At sufficiently low frequencies or close ranges in a calm sea state, $\varrho \left({\bf{r}},\tau | {{\bf{r}}}_{{\bf{0}}}\right)$ does not fall below 0.8.

Beyond the range of current measured data in the Gulf of Mexico continental-shelf environment (Fig. 1(a)), acoustic temporal coherence is predicted to rapidly decay within a short range (<1 km), then gradually decrease further at longer ranges (Fig. 2(a)). This is because: (i) higher order acoustic modes that affect the acoustic field within short ranges interact more with surface gravity waves and quickly lose their temporal coherence (Eqs. (7), (8)) and (ii) the variance of surface gravity waves’ scatter function density is larger for higher acoustic modes because of their steep grazing angles with respect to the mean sea surface (Supplementary Section S2). Contributions from Doppler spread become increasingly important at longer ranges (Fig. 2(a)). In the same environment, the effects of frequency and range on acoustic temporal coherence timescales are shown in Fig. 2(b). For long-range ocean sensing applications using frequencies of roughly a kilo-hertz and below, the given sea state is expected to have a negligible effect at approximately 10 km range since acoustic coherence loss is less than 20% (Fig. 2(b)). At higher frequencies of 10 kHz and above, both effects of surface waveheight decorrelation and Doppler spread from horizontal surface wave motion reduce acoustic coherence by 20–30% and determine the depth-averaged 0.8-crossing coherence timescale, ${\tau }_{0.8}\simeq 0.1$ second at 1 km range.

**Figure 3:**
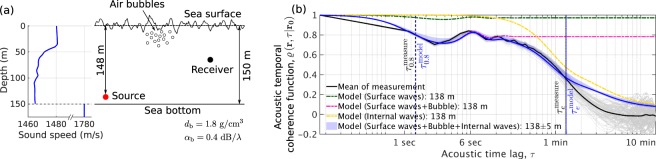
Acoustic temporal coherence loss caused by surface gravity waves, near-sea-surface air bubbles and internal waves in a two-layer continental-shelf environment. These ocean dynamic processes are the dominant mechanisms for coherence loss. Transitions between physical mechanisms that are related to acoustic coherence loss are shown as acoustic time lag increases. (**a**) A Barents Sea continental-shelf environment at a wind speed of 10 m/s (significant waveheight ${H}_{1/3}=2.24$ m and WMO sea state 4) with corresponding bubble densities (Fig. 4(a)) and internal waves of energy density ${E}_{0}=250\,{\rm{J}}/{{\rm{m}}}^{2}$ (Supplementary Table S1). A point source radiating at 240 Hz, 13.82 km from a point receiver at $138\pm 5$ m depth are used to compare predictions with measurements. (**b**) Transitions between dominant acoustic temporal decorrelation mechanisms in the environment described in (**a**). When all three effects are modeled together (blue patch), the predicted acoustic temporal coherence function, $\varrho \left({\bf{r}},\tau | {{\bf{r}}}_{{\bf{0}}}\right)$ (Eq. (3)), is consistent with the measurement (gray solid lines). Sea surface agitation caused by gravity waves and bubbles decorrelate the acoustic field within several seconds or less (magenta dashed line), where the effects of bubbles are dominant over that of surface waves (green dashed line). Internal waves affect acoustic temporal coherence over a longer timescale of minutes or more (yellow dashed line). Dominant temporal decorrelation mechanisms transition from surface waves and air bubbles to internal waves as acoustic time lag increases. This is because surface waves and air bubbles have much shorter coherence timescales than that of internal waves (Fig. 4(b)). The modeled and measured 0.8-crossing coherence timescales (${\tau }_{0.8}$) are respectively 1.35 and 1.37 seconds and the modeled and measured e-folding coherence timescales (${\tau }_{e}$) are respectively 80.64 and 78.12 seconds. The black solid line shows the mean of 100 acoustic temporal coherence curves (gray solid lines) realized using the power spectrum of the measured acoustic pressure field^49^ (Supplementary Section S4).

**Figure 4:**
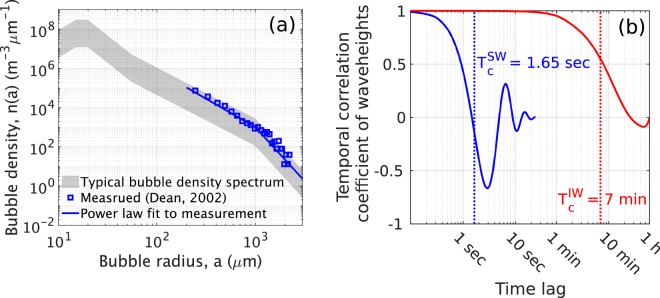
Near-sea-surface air bubble number density spectrum and coherence timescales of the waveheights of surface gravity waves and internal waves. These bubble densities, surface and internal waves are used to predict acoustic temporal coherence loss and attenuation in contiental-shelf environments (Figs. 3(a) and 7(a)). (**a**) Typical bubble number density spectrum of near-sea-surface air bubbles within dense bubble clouds at a wind speed of 10 m/s in an open sea environment. The gray shaded patch shows the range of bubble number densities. Blue open squares show a measured bubble number density spectrum^53^ and the blue solid line is a power law fit to the measurement. (**b**) Coherence timescales of the waveheights of surface gravity waves (blue solid line) at 10 m/s wind speed (significant waveheight ${H}_{1/3}=2.24$ m and WMO sea state 4) and internal waves (red solid line) with average energy density ${E}_{0}=250$ J/m${}^{2}$. The coherence timescale of surface waveheights (${T}_{\,{\rm{c}}}^{{\rm{S}}{\rm{W}}\,}=1.65$ seconds) is several orders of magnitude shorter than that of internal wave waveheights (${T}_{\,{\rm{c}}}^{\mathrm{IW}\,}=7$ minutes).

**Figure 5:**
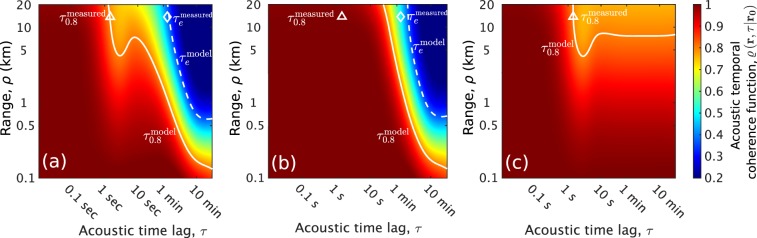
Prediction of the acoustic temporal coherence function, $\varrho \left({\bf{r}},\tau | {{\bf{r}}}_{{\bf{0}}}\right)$ (Eq. (3)), in a two-layer continental-shelf environment (Fig. 3(a)) with varying range and acoustic time lag. At long ranges (>10 km), surface gravity waves and near-sea-surface air bubbles initially reduce acoustic coherence by 20% within seconds. Over longer timescales of minutes, internal waves are the dominant mechanism and cause coherence loss by more than 70%. At short ranges (<1 km), internal waves lead to 70% acoustic coherence loss within ten minutes while the effects of near-sea-surface air bubbles and surface gravity waves are negligible (less than 10% coherence reduction). In (**a**), the effects of surface gravity waves, near-sea-surface air bubbles and internal waves are included, whereas in (**b**) the effects of internal waves are only included. In (**c**), only the effects of surface gravity waves and near-sea-surface air bubbles are included. At ranges of several kilometers or more, surface waves and bubbles reduce acoustic temporal coherence within seconds or less, whereas internal waves decorrelate the acoustic field over several minutes or more. The white solid and dashed lines respectively denote the predicted 0.8-crossing (${\tau }_{0.8}$) and e-folding (${\tau }_{e}$) acoustic temporal coherence timescales. The white open triangle and diamond respectively indicate the measured ${\tau }_{0.8}$ and ${\tau }_{e}$^49^. A wind speed of 10 m/s (significant waveheight ${H}_{1/3}=2.24$ m and WMO sea state 4), corresponding bubble densities (Fig. 4(a)), internal wave energy density ${E}_{0}=250\,{\rm{J}}/{{\rm{m}}}^{2}$, acoustic frequency $f=240$ Hz and 138 m receiver depth are used.

We find a number of dynamic ocean processes affect underwater acoustic propagation and temporal coherence undergoes transitions between the physical mechanisms related to these processes as time lag increases. In particular, these are surface gravity waves, near-sea-surface air bubbles and internal waves. This is investigated in a typical two-layer continental-shelf environment in the Barents Sea at low frequency and high sea state (wind speed 10 m/s, significant waveheight ${H}_{1/3}=2.24$ m, WMO sea state 4, Fig. 3(a)). Dense bubble clouds (Fig. 4(a)) typically form by breaking waves in this high sea state and strong internal wave activities (internal wave energy ${E}_{0}=250$ J/m${}^{2}$) are expected in this two-layer continental shelf environment. Predicted and measured^49^ acoustic temporal coherence functions are consistent across time lag, as shown in Fig. 3(b). Acoustic temporal coherence (Eq. (3)) is predicted from Eq. (2) using the modal coefficients of field-temporal-covariance (Eqs. (7) and (8)) and attenuation (Eqs. (5) and (6)) for each dynamic ocean process (Supplementary Sections S2, S3 and ref. ^25^). At long ranges greater than ten kilometers, surface gravity waves and near-sea-surface air bubbles are found to reduce acoustic temporal coherence by roughly 20% within a relatively short timescale of seconds or less and determine the 0.8-crossing coherence timescale, ${\tau }_{0.8}$ (Fig. 3(b)). Within this short timescale, near-sea-surface bubbles are dominant (Fig. 3(b)). Over long timescales of tens of seconds to minutes, internal waves are found to be the dominant cause of acoustic temporal coherence loss of 70% or more and determine the e-folding coherence timescale, ${\tau }_{e}$ (Fig. 3(b)). This transition between mechanisms occurs because surface waveheight, bubble size and number density vary over much shorter timescales than internal wave compressibility and density fluctuations (Fig. 4(b)). As range decreases to within a kilometer, however, surface gravity waves and near-sea-surface air bubbles are predicted to have a negligible effect (less than 10% coherence reduction, Fig. 5(c)) on low-frequency acoustic temporal coherence, whereas internal waves retain their significant effects within timescales of tens of minutes (Fig. 5(b)).

**Figure 6:**
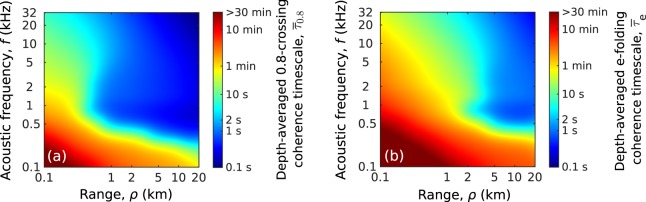
Prediction of the depth-averaged (**a**) 0.8-crossing (${\bar{\tau }}_{0.8}$) and (**b**) e-folding (${\bar{\tau }}_{e}$) acoustic coherence timescales in a two-layer continental-shelf environment (Fig. 3(a)) at general ranges and frequencies for ocean sensing and communication. Surface gravity waves, near sea-surface air bubbles and internal waves are the dominant mechanisms for coherence loss. At frequencies of 0.2–10 kHz and ranges greater than 1 km, surface gravity waves and near-sea-surface air bubbles reduce acoustic temporal coherence by 20–50% within 10 seconds or less and determine ${\bar{\tau }}_{0.8}$. At these frequencies and ranges, internal waves continue to lower the coherence by more than 70% over a longer timescale of 10 minutes or less and determine ${\bar{\tau }}_{e}$. At higher frequencies above 10 kHz and ranges beyond 1 km, surface gravity waves and near-sea-surface air bubbles cause acoustic coherence loss of more than 70% within ten seconds or less and determine both ${\bar{\tau }}_{0.8}$ and ${\bar{\tau }}_{e}$. At these high frequencies, ${\bar{\tau }}_{0.8}$ is less than a second at ranges greater than 2 km and ${\bar{\tau }}_{e}$ falls below a second at ranges beyond 5 km. A wind speed of 10 m/s (significant waveheight ${H}_{1/3}=2.24$ m and WMO sea state 4), corresponding bubble densities (Fig. 4(a)) and internal wave energy density ${E}_{0}=250\,{\rm{J}}/{{\rm{m}}}^{2}$ are used.

Beyond the range of measured data in the Barents Sea (Fig. 3(a)), the effects of surface gravity waves, near-sea-surface air bubbles and internal waves on acoustic coherence timescales are shown in Fig. 6. The given high sea state and strong internal waves are expected to significantly affect acoustic propagation for many frequencies and ranges relevant to sensing and communication. At frequencies of 0.2–10 kHz and ranges greater than 1 km, transitions between dominant mechanisms that reduce acoustic temporal coherence are expected. At these frequencies and ranges, surface gravity waves and near-sea-surface air bubbles reduce acoustic temporal coherence by 20–50% within 10 seconds or less and determine the depth-averaged 0.8-crossing coherence timescale, ${\bar{\tau }}_{0.8}$ (Fig. 6(a)). Internal waves continue to decrease the coherence by more than 70 % over a longer timescale of 10 minutes or less and determine the depth-averaged e-folding coherence timescale, ${\bar{\tau }}_{e}$ (Fig. 6(b)). At higher frequencies above 10 kHz and ranges beyond 1 km, however, this transitions between physical mechanisms do not occur. Surface gravity waves and near-sea-surface air bubbles cause acoustic coherence loss of more than 70% within ten seconds or less and determine both ${\bar{\tau }}_{0.8}$ and ${\bar{\tau }}_{e}$. At these high frequencies, ${\bar{\tau }}_{e}$ falls below a second at ranges greater than 5 km (Fig. 6(b)), which is consistent with coherence timescales often measured in typical underwater communication channels^50^.

We find that the lengths of whale vocalizations measured over great distances^11^ are shorter than the predicted coherence timescales at typical continental-shelf environments even in high sea states (Fig. 3). The whale calls measured at these long ranges (>10 km)^11^ are typically 0.05–2.33 seconds long depending on the species. Their center frequencies vary between 20 Hz and 2.7 kHz^11^. At these ranges and frequencies, the predicted coherence timescale varies between 1 second and 2.5 minutes (Fig. 6(b)), which is comparable or longer than the length of the measured whale vocalizations. This is possibly an adaptation to the environment and is consistent with the calls being used intentionally to carry over great distances without distortion from temporal fluctuations in the medium. Deterministic waveguide dispersion will occur over these short timescales for long-range propagation, however, this can be recognized^12^ or deconvolved^51^.

### Acoustic attenuation by surface gravity waves and near-sea-surface air bubbles

**Figure 7:**
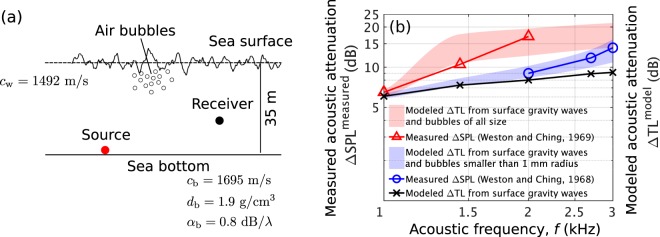
Acoustic attenuation caused by surface gravity waves and near-sea-surface air bubbles in an isovelocity continental shelf environment. Bubbles become the dominant mechanism as frequency increases. Large bubbles of radii larger than 1 mm especially lead to significant attenuation despite their low number density as the acoustic transmission frequency approaches the resonant scattering frequency of the large bubbles. (**a**) A Bristol Channel continental-shelf environment at a wind speed of 10 m/s (significant waveheight ${H}_{1/3}=2.24$ m and WMO sea state 4) with corresponding bubble densities (Fig. 4(a)) (Supplementary Table S1). A point source radiating at 1, 1.44, 2, 2.7 and 3 kHz, 23 km from a point receiver mounted at the sea floor are used to compare predictions with measurements. (**b**) Comparison between predicted ($\Delta {\mathrm{TL}}^{\mathrm{model}}$: Eq. (10)) and measured ($\Delta {\mathrm{SPL}}^{\mathrm{measured}}$: Eq. (9)) acoustic attenuation by surface gravity waves and near-sea-surface air bubbles in the environment described in (**a**). Shaded patches are the modeled acoustic attenuation caused both by surface gravity waves and near-sea-surface air bubbles. The red shaded patch shows attenuation including the effects of bubbles of all size, whereas the blue shaded patch shows attenuation when only bubbles smaller than 1 mm radius are included. These predictions are consistent with acoustic attenuation measurements^15^ that are denoted as red triangles and blue circles. The predicted acoustic attenuation caused by surface gravity waves is shown as a black solid line with cross markers at corresponding measurement frequencies. Upper and lower limits of each shaded patch corresponds to the upper and lower bounds of a typical bubble number density, as shown by the gray shaded patch in Fig. 4(a).

Here, we quantify acoustic attenuation caused by sea-surface agitation in an isovelocity continental-shelf environment in the Bristol Channel at high sea state (wind speed 10 m/s, significant waveheight ${H}_{1/3}=2.24$ m, WMO sea state 4, Fig. 7(a)) in the mid-frequency range of roughly 0.5–5 kHz. Internal wave effects are insignificant in this environment because the medium sound speed is constant^15^. Modeled (Section 2.1) and measured^15^ acoustic attenuation caused by surface gravity waves and near-sea-surface air bubbles are found to be consistent throughout the entire frequency range investigated (Fig. 7(b)). Bubbles are found to become important as frequency increases (Fig. 7(b)). Large bubbles of radii greater than 1 mm, in particular, are shown to have significant effects (Fig. 7(b)) compared to more numerous micro-bubbles (bubbles of radii smaller than 1 mm) (Fig. 4(a)) because the resonance scattering frequency of these large bubbles is near the acoustic transmission frequency.

Acoustic attenuation due to sea-surface disturbances is measured^15^ by the Sound Pressure Level (SPL) decrease in high sea states with respect to the SPL measured in calm sea states as shown in Fig. 7(b). This can be expressed as

9 $$\Delta {{\rm{S}}{\rm{P}}{\rm{L}}}^{{\rm{m}}{\rm{e}}{\rm{a}}{\rm{s}}{\rm{u}}{\rm{r}}{\rm{e}}{\rm{d}}}={{\rm{S}}{\rm{P}}{\rm{L}}}^{{\rm{m}}{\rm{e}}{\rm{a}}{\rm{s}}{\rm{u}}{\rm{r}}{\rm{e}}{\rm{d}}}\,({\bf{r}}|{{\bf{r}}}_{0};U < 5\,{\rm{m}}/{\rm{s}})-{{\rm{S}}{\rm{P}}{\rm{L}}}^{{\rm{m}}{\rm{e}}{\rm{a}}{\rm{s}}{\rm{u}}{\rm{r}}{\rm{e}}{\rm{d}}}\,({\bf{r}}|{{\bf{r}}}_{0};U),$$

where ${\mathrm{SPL}}^{\mathrm{measured}}\ ({\bf{r}}| {{\bf{r}}}_{0};U < 5\,{\rm{m}}/{\rm{s}}\,)$ is the SPL measured at a calm sea state of wind speeds below 5 m/s and ${\mathrm{SPL}}^{\mathrm{measured}}\ ({\bf{r}}| {{\bf{r}}}_{0};U)$ denotes the SPL measured at relatively high sea states of wind speeds above 5 m/s. By using the measured $\,\mathrm{SPL}\,$ at low wind speeds (<5 m/s) as a reference in Eq. (9), acoustic attenuation caused by sea-surface agitations is isolated by removing the attenuation due to extraneous mechanisms such as scattering from the sea bottom. The isolated effects of inhomogeneities near the sea surface on acoustic attenuation shown in Fig. 7(b) is modeled as

10 $$\Delta {{\rm{T}}{\rm{L}}}^{{\rm{m}}{\rm{o}}{\rm{d}}{\rm{e}}{\rm{l}}}={{\rm{T}}{\rm{L}}}^{{\rm{m}}{\rm{o}}{\rm{d}}{\rm{e}}{\rm{l}}}\,({\bf{r}}|{{\bf{r}}}_{0};U=5\,{\rm{m}}/{\rm{s}})-{{\rm{T}}{\rm{L}}}^{{\rm{m}}{\rm{o}}{\rm{d}}{\rm{e}}{\rm{l}}}\,({\bf{r}}|{{\bf{r}}}_{0};U,{N}_{0}).$$

Here, the modeled Transmission Loss (TL) is defined as $\,\mathrm{TL}\,=10{\log }_{10}\langle {| {\Phi }_{T}({\bf{r}}| {{\bf{r}}}_{0})| }^{2}\rangle$, which can be calculated using Eq. (2) with zero acoustic time lag. Acoustic attenuation is predicted by taking the difference between the modeled TL that only includes the effects of surface gravity waves at 5 m/s wind speed, ${\mathrm{TL}}^{\mathrm{model}}\ ({\bf{r}}| {{\bf{r}}}_{0};U=5\,{\rm{m}}/{\rm{s}})$, and the modeled TL that includes both effects of surface gravity waves and near-sea-surface air bubbles at higher wind speeds, ${\mathrm{TL}}^{\mathrm{model}}\ ({\bf{r}}| {{\bf{r}}}_{0};U,{N}_{0})$, where ${N}_{0}$ is the bubble number spectral density at a bubble radius of 100 $\mu$m. At 5 m/s wind speed, bubble effects on acoustic propagation within the investigated frequency range are negligible because of the insufficient number of bubbles that form at such low sea states^52^.

Seasonal variations in 1968 and 1969 attenuation measurements^15^ can be quantitatively explained by fluctuations in bubble formation. This effect was suggested in speculations by Weston^15^ and quantitatively confirmed here as shown in Fig. 7(b). Although the sea states were similar in both experiments, seasonal changes in water column stability can affect the level of turbulent fragmentation that generates bubbles with radii larger than the Hinze scale^53^ (1 mm).

**Figure 8:**
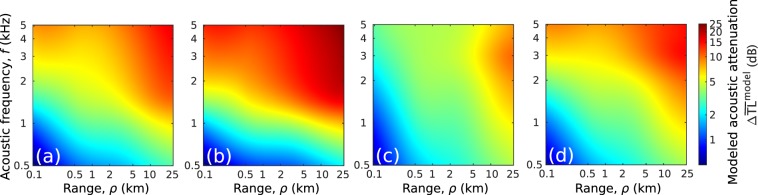
Prediction of the depth-averaged acoustic attenuation caused by surface gravity waves and near-sea-surface air bubbles in an isovelocity continental shelf environment (Fig. 7(a)) with varying range and frequency. The acoustic field rapidly attenuates within short ranges (<1 km), and continues to gradually attenuate over longer ranges (>1 km) because the higher order acoustic modes interact more with the ocean inhomogeneities and attenuate within a short distance. Bubbles of radii larger than 1 mm are expected to have a significant effect on acoustic attenuation at the shown frequency range in spite of their much lower number density than micro bubbles of radii smaller than 1 mm. (**a**) The lower limit of a plausible bubble number density spectrum that includes all bubble sizes (Lower bound of the gray patch shown in Fig. 4(a)) is used for attenuation prediction. (**b**) Same as (**a**), but the upper limit of the plausible bubble number density spectrum that includes all bubble sizes (Upper bound of the gray patch shown in Fig. 4(a)) is used. (**c**) Same as (**a**), but only including the number density spectrum for bubbles of radii smaller than 1 mm. (**d**) Same as (**b**), but only including the number density spectrum for bubbles of radii smaller than 1 mm. A wind speed of 10 m/s (significant waveheight ${H}_{1/3}=2.24$ m and WMO sea state 4) and corresponding bubble densities (Fig. 4(a)) are used.

Beyond the range of measured data in the Bristol Channel environment (Fig. 7(a)), attenuation due to surface gravity waves and near-sea-surface air bubbles rapidly accumulates within short ranges (<1 km), and continues to gradually accumulate over longer ranges (>1 km) (Fig. 8). This is again because the higher order acoustic modes that affect the acoustic field within short ranges interact more with surface gravity waves and near-sea-surface air bubbles and attenuate rapidly (Eqs. (7), (8)). At longer ranges where higher order acoustic modes are sufficiently attenuated, lower order acoustic modes gradually attenuate since they interact less with the inhomogeneities.

## Discussion and Conclusions

An analytic theory for the combined effects of dynamic oceanic processes on acoustic temporal coherence and attenuation has been derived. This has enabled the dominant physical mechanisms to be identified as a function of frequency, range, acoustic coherence timescale and oceanographic conditions for a broad range of sensing and communication applications in the ocean. These dominant mechanisms are found to be surface gravity waves, near-sea-surface air bubbles and internal waves. The expressions are derived from first principles and include Doppler effects in an ocean waveguide containing 3-D moving random inhomogeneities. The approach requires only standard oceanographic measurements for acoustic predictions, such as sea state, particle velocity at the sea surface, bubble number density or internal wave energy. The current study quantifies the combined effects of various ocean dynamic processes on acoustic propagation and enables transitions between dominant physical mechanisms to be predicted.

Here we find degradations in acoustic temporal coherence are caused both by inhomogeneous fluctuations in the ocean medium as well as Doppler spread caused by the translation of these inhomogeneities. Previously, the coherence timescale of a signal has often been expressed as the reciprocal of Doppler spread^30,54,55^, which implies coherence degradation is due solely to relative motion between the source, receiver and inhomogeneities in the medium. We find that this is only the case for relatively high-frequency and short-range acoustic sensing or communication scenarios in the ocean. For long-range ocean applications where frequencies are typically lower due to volumetric absorption issues^31^, we find acoustic coherence times are determined by the timescale of the processes that cause spatial and temporal fluctuations in the medium.

It is necessary for acoustic signals to be coherent over a specific time in many applications of sensing and communication in the ocean including standard image formation by beamforming as well as signal detection, identification, processing and decoding after matched filtering which both rely on coherent temporal signal patterns. Here we quantify the maximum time period expected for acoustic signals transmitted in the ocean to be coherent given the limiting effects of natural oceanic fluctuations. This timescale, for example, is a factor that bounds the maximum coherent processing gain available in communication and matched filtering, and also determines the number of independent samples for statistical variance reduction via stationary averaging over a given measurement window^19^ in typical ocean sensing scenarios^4,5^. The number of independent samples in a given measurement time is the measurement time divided by the acoustic temporal coherence time, given stationarity, and leads to variance reduction by a factor of the number of independent samples. It is known that many marine creatures use sound to communicate and sense their environment. Marine mammal vocalizations that carry over great distances, which are natural acoustic signals that have been associated with communication and sensing, are found to have typical durations that are consistent with the coherence timescales quantified here. This may be related to prevention of random distortion of a signal that may interfere with its information content and recognition even by temporally incoherent reception.

## Supplementary information

Supplementary Information.

## Data Availability

Simulated data of this study are included in the current article and its supplementary information. Measured data are available at the cited publications.
