# Supplementary material for: Predicting the Effects of Random Ocean Dynamic Processes on Underwater Acoustic Sensing and Communication
Source: Sci Rep. 2020 Mar 11;10:4525. doi: 10.1038/s41598-020-61043-w (PMC7066198; doi:10.1038/s41598-020-61043-w)
Supplement: Supplementary file 1 — Supplementary Information. [file 41598_2020_61043_MOESM1_ESM.pdf]

Supplementary Information for Predicting the Effects of Random Ocean Dynamic Processes on Underwater Acoustic Sensing and Communication

Byunggu Cho[1] **and Nicholas C. Makris**[1,*]

[1] Center for Ocean Engineering, Massachusetts Institute of Technology, 77 Mass. Ave., Cambridge MA 02139
[*] makris@mit.edu

S1 Mean and Temporal Covariance of the Acoustic Field Forward Propagated Through Moving Random Inhomogeneities in a 3-D Waveguide

In this section, we provide a full derivation of analytic expressions for the mean and temporal covariance of the acoustic forward field propagated through moving random inhomogeneities in a 3-D waveguide. We extend previous formulations[1–3] by including Doppler effects caused by the inhomogeneities' motion in the waveguide. These earlier analytic expressions were developed for the mean and temporal covariance of the acoustic field forward propagated through a 3-D ocean waveguide containing motionless random inhomogeneities. They were confirmed by a number of experimental measurements and shown to predict and quantify the effects of random internal waves on acoustic forward field in a waveguide[2–4]. Chen et al.[2] showed that the acoustic forward field becomes effectively incoherent at typical operational ranges when the root-mean-square internal wave height is on the order of the acoustic wavelength and that two-dimensional models for the mean and variance of the acoustic field propagated through 3-D random internal waves become inaccurate when the Fresnel width approaches and exceeds the cross-range coherence length of the internal waves. Chen et al.[3] also predicted the coherence time scale of acoustic forward field fluctuations caused by internal waves in the deep ocean and explained the time scale of acoustic field fluctuations observed at mega-meter ranges in various deep-ocean acoustic transmission experiments. Gong et al.[4] predicted the temporal coherence of the acoustic field forward propagated through a continental-shelf waveguide containing internal waves as a function of internal wave energy and confirmed the predictions with various acoustic measurements.

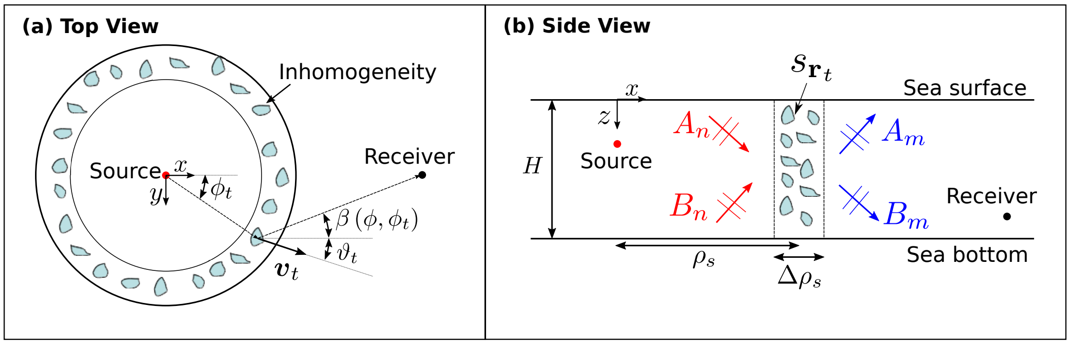

Figure S1. (a) Top and (b) side views of the coordinate system and configurations of the source, receiver and inhomogeneities within a single cylindrical differential range slab.

S1.1 Analytic Expression for the Mean Forward Field

Scattered field from a differential range slab of radius ρ_s and thickness $\Delta\rho_s$ containing moving inhomogeneities (Figure S1) can be expressed as

$$\Phi_s(\mathbf{r},t|\mathbf{r_0};\Delta\rho_s(\rho_s)) = \iiint\limits_{\Delta V_s} d\mathbf{r_t^0}\,\varphi_s(\mathbf{r},t|\mathbf{r_0};\mathbf{r_t^0}), \tag{S1}$$

where $\varphi_s(\mathbf{r},t|\mathbf{r_0};\mathbf{r_t^0})$ is the scattered field per unit volume and $\Delta V_s = 2\pi\rho_s\Delta\rho_s H$ is the volume of a differential range slab as shown in Figure S1. The origin of the coordinate system is at the water-atmosphere interface directly above the source and the positive z direction is downward. The receiver coordinate $(\mathbf{r},\mathrm{t}) = (x,y,z,\mathrm{t}) = (\rho,\phi,z,\mathrm{t})$, where (x,y,z) and (ρ,ϕ,z) are

respectively the expressions in Cartesian and Cylindrical coordinate systems and t is the receiver time. Coordinate of an inhomogeneity is $(\mathbf{r_t}(\mathrm{t}_t),\mathrm{t}_t) = (x_t(\mathrm{t}_t), y_t(\mathrm{t}_t), z_t(\mathrm{t}_t), \mathrm{t}_t) = (\rho_t(\mathrm{t}_t), \phi_t(\mathrm{t}_t), z_t(\mathrm{t}_t), \mathrm{t}_t)$, where the position of an inhomogeneity, $\mathbf{r_t}(\mathrm{t}_t)$, is a function of time, t_t, since the inhomogeneities are moving. The position of an inhomogeneity at an initial time at $\mathrm{t}_t = 0$ is denoted as $\mathbf{r_t}(\mathrm{t}_t = 0) = \mathbf{r_t^0} = \left(x_t^0, y_t^0, z_t^0\right) = \left(\rho_t^0, \phi_t^0, z_t^0\right)$. The source positioned directly below the origin of the coordinate system at $\mathbf{r_0} = (x_0, y_0, z_0) = (0, 0, z_0)$ operates at a single acoustic frequency ω_i. A modal formulation for the scattered field per unit volume from inhomogeneities at $\mathbf{r_t^0}$ and horizontally moving at a speed slower than the sound speed[5] is

$$
\begin{aligned}
\varphi_s(\mathbf{r}, t|\mathbf{r_0};\mathbf{r_t^0}) = & \sum_n \sum_m e^{-i\omega_{nm}t} \frac{(4\pi)^2}{k(\omega_{nm})} \left(1 + \frac{v_{\mathbf{r_t^0}}}{C_m^g(\omega_{nm})} \cos\left(\vartheta_{\mathbf{r_t^0}} - \beta(\phi, \phi_t^0)\right)\right) \\
& \times \Big[A_m\left(\mathbf{r} - \mathbf{r_t^0}; \omega_{nm}\right) A_n\left(\mathbf{r_t^0} - \mathbf{r_0}; \omega_i\right) s_{\mathbf{r_t^0}}\left(\pi - \alpha_m(\omega_{nm}), \beta(\phi, \phi_t^0); \alpha_n(\omega_i), \phi_t^0; \omega_{nm}\right) \\
& - B_m\left(\mathbf{r} - \mathbf{r_t^0}; \omega_{nm}\right) A_n\left(\mathbf{r_t^0} - \mathbf{r_0}; \omega_i\right) s_{\mathbf{r_t^0}}\left(\alpha_m(\omega_{nm}), \beta(\phi, \phi_t^0); \alpha_n(\omega_i), \phi_t^0; \omega_{nm}\right) \\
& - A_m\left(\mathbf{r} - \mathbf{r_t^0}; \omega_{nm}\right) B_n\left(\mathbf{r_t^0} - \mathbf{r_0}; \omega_i\right) s_{\mathbf{r_t^0}}\left(\pi - \alpha_m(\omega_{nm}), \beta(\phi, \phi_t^0); \pi - \alpha_n(\omega_i), \phi_t^0; \omega_{nm}\right) \\
& + B_m\left(\mathbf{r} - \mathbf{r_t^0}; \omega_{nm}\right) B_n\left(\mathbf{r_t^0} - \mathbf{r_0}; \omega_i\right) s_{\mathbf{r_t^0}}\left(\alpha_m(\omega_{nm}), \beta(\phi, \phi_t^0); \pi - \alpha_n(\omega_i), \phi_t^0; \omega_{nm}\right) \Big],
\end{aligned} \quad \text{(S2)}
$$

where the doubly Doppler-shifted frequency ω_{nm} is

$$
\omega_{nm} \approx \omega_i + \left(\xi_m(\omega_i)\cos\left(\vartheta_{\mathbf{r_t^0}} - \beta(\phi, \phi_t^0)\right) - \xi_n(\omega_i)\cos\left(\vartheta_{\mathbf{r_t^0}} - \phi_t^0\right)\right) v_{\mathbf{r_t^0}}. \quad \text{(S3)}
$$

As shown in Figure S1(a), $v_{\mathbf{r_t^0}}$ is the magnitude of a moving inhomogeneity's horizontal velocity and $\vartheta_{\mathbf{r_t^0}}$ is the azimuth of a moving inhomogeneity's horizontal velocity with respect to the forward direction. $C_m^g(\omega_{nm})$ is the mth modal group velocity evaluated at the Doppler-shifted frequency, ω_{nm}. $A_n\left(\mathbf{r_t^0} - \mathbf{r_0}; \omega_i\right)$ and $B_n\left(\mathbf{r_t^0} - \mathbf{r_0}; \omega_i\right)$ are the *down-* and *up-going* incident modal plane wave components evaluated at the incident frequency ω_i as defined in Equations (3) and (4) of Reference[1]. $A_m\left(\mathbf{r} - \mathbf{r_t^0}; \omega_{nm}\right)$ and $B_m\left(\mathbf{r} - \mathbf{r_t^0}; \omega_{nm}\right)$ are the *up-* and *down-going* scattered modal plane wave components evaluated at the Doppler-shifted frequency ω_{nm} as defined in Equations (5) and (6) of Reference[1]. $s_{\mathbf{r_t^0}}(\omega_{nm})$ is the scatter function density of an inhomogeneity at $\mathbf{r_t^0}$ evaluated at ω_{nm}. The rest of the arguments of $s_{\mathbf{r_t^0}}$ denote the dependency on the propagation directions of the modal incident and scattered plane wave components. $\alpha_n(\omega_i)$ and $\pi - \alpha_n(\omega_i)$ respectively denote the elevation angles of *down-* and *up-going* incident n^{th} modal plane wave components measured from the positive z-axis evaluated at the incident frequency, ω_i. For example, the elevation angle of the nth down-going incident modal component measured from the positive z-axis can be calculated as $\alpha_n(\omega_i) = \mathrm{atan}\left(\Re\{\xi_n(\omega_i)\}/\Re\{\gamma_n(\omega_i)\}\right)$ where $\xi_n(\omega_i)$ and $\gamma_n(\omega_i)$ are the respective n^{th} modal horizontal and vertical wavenumbers at incident frequency, ω_i. Here, $0 \leq \alpha_n \leq \pi/2$ and accordingly the elevation angle of the up-going incident modal component is $\pi - \alpha_n(\omega_i)$. Similarly, $\pi - \alpha_m(\omega_{nm})$ and $\alpha_m(\omega_{nm})$ respectively denote the elevation angles of the *up-* and *down-going* scattered modal plane wave components evaluated at the Doppler-shifted frequency, ω_{nm}. ϕ_t^0 denotes the azimuth of the inhomogeneity and $\beta(\phi, \phi_t^0)$ denotes the receiver azimuth from the inhomogeneity, as shown in Figure S1(a).

A modal formulation for the scattered field from a single moving target derived in Reference[5] is generalized for a slab of moving random inhomogeneities as shown in Equations (S1) and (S2). These inhomogeneities are assumed to move at a speed much slower than the sound speed, which is valid for a wide range of ocean environmental inhomogeneities. Only the horizontal motion of the inhomogeneities is considered because Doppler effects caused by the inhomogeneities' vertical motion is relatively small. This is because the horizontal modal wavenumber, ξ_n, is much greater than the vertical modal wavenumber, γ_n, for propagating acoustic modes. Lai and Makris[5] derived spectral and modal formulations for the scattered field when the source, target and receiver are horizontally moving at a low Mach number in a horizontally stratified waveguide. They showed that the scattered field from a moving target in a waveguide is doubly Doppler-shifted at multiple frequencies because of multi-modal propagation even when the incident field is harmonic. A monochromatic source that excites N incident modes in a waveguide couple with the N outgoing modes through scattering from a moving target and leads to N^2 distinct harmonic components in the scattered field. This scattered field by a moving target in a waveguide is doubly Doppler-shifted in all directions including the forward direction because the off-diagonal ($n \neq m$) acoustic modes couple through scattering. In free space, forward scattering through moving inhomogeneities does not cause Doppler spread as it does in a waveguide because there is no off-diagonal mode-coupling[5]. For random inhomogeneities, where their scatter function density, $s_{\mathbf{r_t^0}}$, and horizontal velocity, $v_{\mathbf{r_t^0}}$, follow a stationary random process horizontally in space within a single differential range slab, the mean forward scattered field, i.e. the expectation of Equation (S1), can be analytically evaluated as

$$
\begin{aligned}
&\langle \Phi_s(\mathbf{r},t|\mathbf{r_0};\Delta\rho_s(\rho_s))\rangle \\
&= \sum_n e^{-i\omega_i t}\Phi_i^{(n)}(\mathbf{r}|\mathbf{r_0}) \times i\sum_m \frac{1}{\xi_m}\frac{u_m(z)}{u_n(z)}\Bigg\langle \int_0^H dz_t^0 e^{-i(\xi_m-\xi_n)\left[v_{z_t^0}\cos(\vartheta_{z_t^0})t-\left(1+v_{z_t^0}\cos\vartheta_{z_t^0}/C_m^g\right)(\rho-\rho_s)\right]} \\
&\times \operatorname{sinc}\left((\xi_m-\xi_n)\left(1+\frac{v_{z_t^0}}{C_m^g}\cos\vartheta_{z_t^0}\right)\frac{\Delta\rho_s}{2}\right)\frac{2\pi}{k(z_t^0)d(z_t^0)}\left(1+\frac{v_{z_t^0}}{C_m^g}\cos\vartheta_{z_t^0}\right) \\
&\times \Big\{ N_m^{(1)}(z_t^0)N_n^{(1)}(z_t^0)e^{i\Re\{\gamma_m(z_t^0)+\gamma_n(z_t^0)\}z_t^0} s_{z_t^0}(\pi-\alpha_m(\omega_{nm}),\phi;\alpha_n(\omega_i),\phi;\omega_{nm}) \\
&- N_m^{(2)}(z_t^0)N_n^{(1)}(z_t^0)e^{i\Re\{-\gamma_m(z_t^0)+\gamma_n(z_t^0)\}z_t^0} s_{z_t^0}(\alpha_m(\omega_{nm}),\phi;\alpha_n(\omega_i),\phi;\omega_{nm}) \\
&- N_m^{(1)}(z_t^0)N_n^{(2)}(z_t^0)e^{i\Re\{\gamma_m(z_t^0)-\gamma_n(z_t^0)\}z_t^0} s_{z_t^0}(\pi-\alpha_m(\omega_{nm}),\phi;\pi-\alpha_n(\omega_i),\phi;\omega_{nm}) \\
&+ N_m^{(2)}(z_t^0)N_n^{(2)}(z_t^0)e^{i\Re\{-\gamma_m(z_t^0)-\gamma_n(z_t^0)\}z_t^0} s_{z_t^0}(\alpha_m(\omega_{nm}),\phi;\pi-\alpha_n(\omega_i),\phi;\omega_{nm}) \Big\} \Bigg\rangle_{\rho_s} \\
&\times e^{-\Im\{\gamma_m(z_t^0)+\gamma_n(z_t^0)\}z_t^0}\Delta\rho_s,
\end{aligned}
\tag{S4}
$$

where stationary phase approximation is used to analytically conduct the integration as described in Reference[1]. This stationary phase analysis shows that only inhomogeneities within the local Fresnel width ($Y_F = \sqrt{\lambda\rho_s(\rho-\rho_s)/\rho}$ where λ is the acoustic wavelength) in cross-range contribute to the forward scattered field. Since $\phi_t \approx 0$ and $\beta(\phi,\phi_t^0) \approx 0$ for the inhomogeneities within Y_F in cross-range, the Doppler shifted frequency, ω_{nm}, in the forward scattered field can be simplified as

$$
\omega_{nm} \approx \omega_i + (\xi_m(\omega_i)-\xi_n(\omega_i))\, v_{z_t^0}\cos\vartheta_{z_t^0}. \tag{S5}
$$

In the forward scattered field, frequency shift occurs only for inhomogeneities with non-zero velocity in the forward direction, and when the incident and scattered modal components are different, i.e. $n \neq m$. In Equation (S4), $\Phi_i^{(n)}(\mathbf{r}|\mathbf{r_0})$ is the nth modal component of the incident field as expressed in Equation (S10). $N_n^{(1)}(z)$ and $N_n^{(2)}(z)$ are respectively the *down-* and *up-going* incident modal amplitudes of an incident mode function $u_n(z) = N_n^{(1)}(z)e^{i\gamma_n(z)z} - N_n^{(2)}(z)e^{-i\gamma_n(z)z}$. Similarly, $N_m^{(1)}(z)$ and $N_m^{(2)}(z)$ are respectively the *up-* and *down-going* scattered modal amplitudes of a scattered mode function $u_m(z) = N_m^{(1)}(z)e^{i\gamma_m(z)z} - N_m^{(2)}(z)e^{-i\gamma_m(z)z}$. As the forward scattered field is integrated over the slab volume with sufficient range thickness, Doppler shift due to the movement of inhomogeneities for different incident and scattered modes are negligible because the off diagonal terms are scaled by $\operatorname{sinc}\left((\xi_m-\xi_n)\left(1+v_{z_t^0}\cos\vartheta_{z_t^0}/C_m^g\right)\Delta\rho_s/2\right) \approx \delta_{nm}$. For the same incident and scattered modes, $n = m$, extension (contraction) in the wavelength of the incident mode cancels the contraction (extension) in the wavelength of the scattered mode, which leads to zero Doppler shift as shown in Equation (S5). Therefore, the double modal sum in Equation (S4) reduces to a single modal sum and all the variables that were evaluated at the Doppler-shifted frequency, ω_{nm}, in Equation (S2) can be evaluated at the incident frequency, ω_i. Then, the mean forward scattered field by a differential range slab of moving inhomogeneities is

$$
\langle \Phi_s(\mathbf{r}|\mathbf{r_0};\Delta\rho_s(\rho_s))\rangle = \sum_n \Phi_i^{(n)}(\mathbf{r}|\mathbf{r_0})\, i\nu_n(\rho_s)\Delta\rho_s, \tag{S6}
$$

where $\nu_n(\rho_s)$ is the modal complex wavenumber change. The real part of the modal complex wavenumber change is evaluated by keeping only the diagonal components of Equation (S4) due to the modal decoupling for a sufficiently large differential range slab[1]. The imaginary part the modal complex wavenumber change is calculated by Equation (S13) in regions where 2-D scattering occurs and by Equation (S14) where 3-D scattering occurs. Since the mean forward scattered field from a differential range slab of moving inhomogeneities is equal to the change in the mean forward total field, we can formulate a difference equation about the mean forward total field as

$$
\langle \Phi_s^{(n)}(\mathbf{r}|\mathbf{r_0};\Delta\rho_s(\rho_s))\rangle = \Delta\langle \Phi_T^{(n)}(\mathbf{r}|\mathbf{r_0})\rangle = \langle \Phi_T^{(n)}(\mathbf{r}|\mathbf{r_0})\rangle i\nu_n(\rho_s)\Delta\rho_s, \tag{S7}
$$

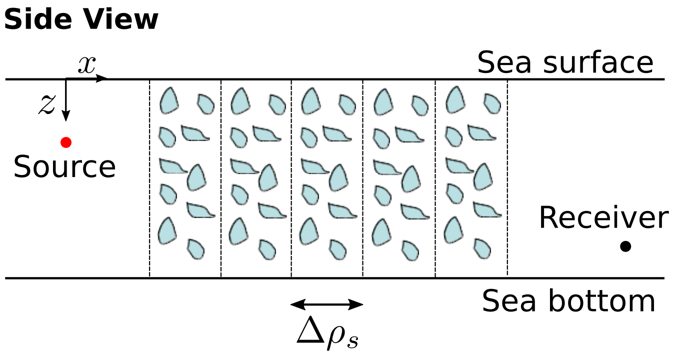

Figure S2. Schematic diagram of marching the statistical moments of acoustic forward field through multiple slabs containing random inhomogeneities.

using Equation (S6). Here, $\langle \Phi_s^{(n)}(\mathbf{r}|\mathbf{r_0};\Delta\rho_s(\rho_s))\rangle$ and $\Delta\langle \Phi_T^{(n)}(\mathbf{r}|\mathbf{r_0})\rangle$ are respectively the nth modal components of the mean forward scattered field and the mean forward total field change caused by a single differential range slab of moving inhomogeneities.

The mean forward total field is then marched through contiguous slabs containing inhomogeneities as described in Figure S2 to include multiple forward scattering from the source to the receiver, which is analogous to a scheme by Rayleigh[6,7] and others[8,9] for free space. This marching scheme is expressed by rewriting Equation (S7) as an integral equation

$$\int_{\psi_i^{(n)}}^{\langle \psi_T^{(n)} \rangle} \frac{d\langle \Phi_T^{(n)}(\mathbf{r}|\mathbf{r_0})\rangle}{\langle \Phi_T^{(n)}(\mathbf{r}|\mathbf{r_0})\rangle} = i\int_0^{\rho} \nu_n(\rho_s)d\rho_s. \tag{S8}$$

Solving Equation (S8), the mean forward total field after propagating through a 3-D waveguide of moving random inhomogeneities can be expressed as

$$\langle \Phi_T(\mathbf{r}|\mathbf{r_0})\rangle = \sum_n \Phi_i^{(n)}(\mathbf{r}|\mathbf{r_0}) \exp\left[i\int_0^{\rho} \nu_n(\rho_s)d\rho_s\right], \tag{S9}$$

where the nth modal component of the incident field, $\Phi_i^{(n)}(\mathbf{r}|\mathbf{r_0})$, is given by

$$\Phi_i^{(n)}(\mathbf{r}|\mathbf{r_0}) = \frac{i\sqrt{2\pi}}{d(z_0)} \frac{e^{i(\xi_n\rho - \frac{\pi}{4})}}{\sqrt{\xi_n\rho}} u_n(z_0)u_n(z). \tag{S10}$$

Here, $u_n(z_0)$ and $u_n(z)$ are respectively the nth incident mode function evaluated at the source and receiver depths. $\nu_n(\rho_s)$ is the nth modal complex wavenumber change at horizontal range ρ_s caused by the moving random inhomogeneities. Intragration in Equation (S9) is numerically evaluated using a finite range slab width, $\Delta\rho_s$, that decouples the distinct modes by satisfying Equation (57) of Reference[1]. The real part of the modal complex wavenumber change, $\Re\{\nu_n(\rho_s)\}$, which accounts for the modal dispersion in the mean acoustic forward field, is expressed as

$$\Re\{\nu_n(\rho_s)\} = \Re\left\{\frac{1}{\xi_n}\int_0^H dz_t^0 \langle F_{z_t^0}(n,n;0,0)\rangle\right\}, \tag{S11}$$

where,

$$
\begin{aligned}
\mathcal{F}_{z_t^0}(m,n;\beta,\beta_i) = & \frac{2\pi}{k(z_t^0)d(z_t^0)}\left(1+\frac{v_{z_t^0}}{C_m^g}\cos\vartheta_{z_t^0}\right) \\
& \times \Big\{ N_m^{(1)}(z_t^0)N_n^{(1)}(z_t^0)e^{i\Re\{\gamma_m(z_t^0)+\gamma_n(z_t^0)\}z_t^0} s_{z_t^0}(\pi-\alpha_m,\beta;\alpha_n,\beta_i) \\
& - N_m^{(2)}(z_t^0)N_n^{(1)}(z_t^0)e^{i\Re\{-\gamma_m(z_t^0)+\gamma_n(z_t^0)\}z_t^0} s_{z_t^0}(\alpha_m,\beta;\alpha_n,\beta_i) \\
& - N_m^{(1)}(z_t^0)N_n^{(2)}(z_t^0)e^{i\Re\{\gamma_m(z_t^0)-\gamma_n(z_t^0)\}z_t^0} s_{z_t^0}(\pi-\alpha_m,\beta;\pi-\alpha_n,\beta_i) \\
& + N_m^{(2)}(z_t^0)N_n^{(2)}(z_t^0)e^{i\Re\{-\gamma_m(z_t^0)-\gamma_n(z_t^0)\}z_t^0} s_{z_t^0}(\alpha_m,\beta;\pi-\alpha_n,\beta_i) \Big\} e^{-\Im\{\gamma_m(z_t^0)+\gamma_n(z_t^0)\}z_t^0}. \qquad \text{(S12)}
\end{aligned}
$$

The difference between Equation (S11) and Equation (60a) of Reference[1] is the amplification factor, $1+v_{z_t^0}\cos\vartheta_{z_t^0}/C_m^g$, caused by the movement of inhomogeneities. Additional attenuation in the forward field by this amplification factor is negligibly small when the inhomogeneities are moving at a low Mach number. Amplified dispersion by the movement of inhomogeneities, however, may have noticeable effects on standard coherent processing, e.g. phase coherent underwater acoustic communication, match filter processing, beamforming or synthetic aperture processing for ocean sensing. Numerical integration over the depth in Equation (S11) is conducted using a finite depth increment that is much smaller than the acoustic wavelength.

Assuming no power loss in the forward direction for a 2-D scattering process, the imaginary part of the modal complex wavenumber change, $\Im\{\nu_n(\rho_s)\}$, which accounts for the modal attenuation in the mean acoustic forward field, can be calculated from Equation (37) of Reference[3] as

$$
\Im\{\nu_n^{2-D}(\rho_s)\} = \frac{1}{2}\mu_n^{2-D}(\rho_s,\tau=0), \qquad \text{(S13)}
$$

where $\mu_n^{2-D}(\rho_s,\tau)$ is given in Equation (S36). For 3-D scattering process, 3-D scattering causes power loss in the forward direction, where $\Im\{\nu_n(\rho_s)\}$ can be calculated from Equation (36) of Reference[2] as

$$
\Im\{\nu_n^{3-D}(\rho_s)\} = \frac{1}{4\pi}\sum_{m=1}^{\infty}\Re\left\{\frac{\xi_m^*}{|\xi_m|\Re\{\xi_n^*\}}\int_0^H dz_t^0\int_0^H dz_{t'}^0 A_c(\rho_s,z_t^0,z_{t'}^0)\int_0^{2\pi}\langle \mathcal{F}_{z_t^0}(m,n,\beta,0)\mathcal{F}_{z_{t'}^0}^*(m,n,\beta,0)\rangle d\beta\right\} \qquad \text{(S14)}
$$

by applying the waveguide extinction theorem[10]. Here $A_c(\rho_s,z_t^0,z_t^{0'})$ is the coherence area of the scatter function density of the inhomogeneities given in Equation (A7) of Reference[1] and $\mathcal{F}_{z_t^0}(m,n;\beta,\beta_i)$ is given in Equation (S12). For the numerical integrations in Equation (S14), a finite depth increment that is much less than the acoustic wavelength is used, while the azimuth increment was chosen differently for each inhomogeneity since their dependence on the azimuth varies. For example, the air bubbles are omni-directional inhomogeneities, while internal wave inhomogeneities and surface gravity waves are directional scatterers.

S1.2 Temporal Covariance of The Forward Field

The total field after scattering from a single differential range slab of inhomogeneities can be expressed as

$$
\Phi(\mathbf{r},\mathrm{t}|\mathbf{r_0}) = \Phi_i(\mathbf{r},\mathrm{t}|\mathbf{r_0}) + \Phi_s(\mathbf{r},\mathrm{t}|\mathbf{r_0};\Delta\rho_s(\rho_s)), \qquad \text{(S15)}
$$

where $\Phi_i(\mathbf{r},\mathrm{t}|\mathbf{r_0})$ is the incident field and $\Phi_s(\mathbf{r},\mathrm{t}|\mathbf{r_0};\Delta\rho_s(\rho_s))$ is the scattered field from moving inhomogeneities within a differential range slab of thickness $\Delta\rho_s$ at range ρ_s. The depth-integrated temporal correlation of this total field at an acoustic time lag of $\tau = \mathrm{t}_1 - \mathrm{t}_2$ can then be expressed as

$$
\begin{aligned}
& \int_0^H dz\frac{1}{d(z)}\langle\Phi(\mathbf{r},\mathrm{t}_1|\mathbf{r_0})\Phi^*(\mathbf{r},\mathrm{t}_2|\mathbf{r_0})\rangle \\
& = \int_0^H dz\frac{1}{d(z)}\Phi_i(\mathbf{r},\mathrm{t}_1|\mathbf{r_0})\Phi_i^*(\mathbf{r},\mathrm{t}_2|\mathbf{r_0}) \\
& + \int_0^H dz\frac{1}{d(z)}\{\Phi_i(\mathbf{r},\mathrm{t}_1|\mathbf{r_0})\langle\Phi_s^*(\mathbf{r},\mathrm{t}_2|\mathbf{r_0};\Delta\rho_s(\rho_s))\rangle + \Phi_i^*(\mathbf{r},\mathrm{t}_2|\mathbf{r_0})\langle\Phi_s(\mathbf{r},\mathrm{t}_1|\mathbf{r_0};\Delta\rho_s(\rho_s))\rangle\} \\
& + \int_0^H dz\frac{1}{d(z)}\langle\Phi_s(\mathbf{r},\mathrm{t}_1|\mathbf{r_0};\Delta\rho_s(\rho_s))\Phi_s^*(\mathbf{r},\mathrm{t}_2|\mathbf{r_0};\Delta\rho_s(\rho_s))\rangle. \qquad \text{(S16)}
\end{aligned}
$$

The first term on the right hand side of Equation (S16) is the temporal correlation function of the incident acoustic power that can be expressed as

$$\int_0^H dz \frac{1}{d(z)} \Phi_i(\mathbf{r}, t_1|\mathbf{r_0}) \Phi_i^*(\mathbf{r}, t_2|\mathbf{r_0}) = e^{-i\omega_i \tau} \sum_n W_i^{(n)}(\rho|\mathbf{r_0}), \tag{S17}$$

where $W_i^{(n)}(\rho|\mathbf{r_0})$ is given by

$$W_i^{(n)}(\rho|\mathbf{r_0}) = \int_0^H dz \frac{1}{d(z)} \left| \Phi_i^{(n)}(\mathbf{r}|\mathbf{r_0}) \right|^2 = \frac{2\pi}{d^2(z_0)} \frac{|u_n(z_0)|^2}{\rho\, |\xi_n|} e^{-2\Im\{\xi_n\}\rho}. \tag{S18}$$

The second term in the right hand side of Equation (S16) is the depth-integrated temporal cross correlation function of the incident and scattered fields. From Equation (S6), this term can be evaluated as

$$\begin{aligned} &\int_0^H dz \frac{1}{d(z)} \{ \Phi_i(\mathbf{r}, t_1|\mathbf{r_0}) \langle \Phi_s^*(\mathbf{r}, t_2|\mathbf{r_0}; \Delta\rho_s(\rho_s)) \rangle + \Phi_i^*(\mathbf{r}, t_2|\mathbf{r_0}) \langle \Phi_s(\mathbf{r}, t_1|\mathbf{r_0}; \Delta\rho_s(\rho_s)) \rangle \} \\ &= -e^{-i\omega_i \tau} \sum_n W_i^{(n)}(\rho|\mathbf{r_0}) 2\Im\{\nu_n(\rho_s)\} \Delta\rho_s. \end{aligned} \tag{S19}$$

The third term in the right hand side of Equation (S16) is the temporal correlation function of the scattered power from the differential range slab of inhomogeneities that can be evaluated as

$$\int_0^H dz \frac{1}{d(z)} \langle \Phi_s(\mathbf{r}, t_1|\mathbf{r_0}) \Phi_s^*(\mathbf{r}, t_2|\mathbf{r_0}) \rangle = e^{-i\omega_i \tau} \sum_n W_i^{(n)}(\rho|\mathbf{r_0}) \mu_n(\rho_s, \tau) \Delta\rho_s, \tag{S20}$$

where $\mu_n(\rho_s, \tau)$ is the modal field-temporal-covariance coefficient given in Equations (S36) and (S35). $\mu_n(\rho_s, \tau)$ represents the energy transfer from the mean field to the covariance field and quantifies the decorrelation of the forward field over time. $\mu_n(\rho_s, \tau)$ depends on the spatial and temporal covariance of the scatter function density and the velocity of the inhomogeneities as discussed in Section S1.3. From Equations (S17), (S19) and (S20), a difference equation that describes the change in the temporal correlation of the total acoustic power due to scattering from the inhomogeneities in a differential range slab can be expressed as

$$\Delta\langle W_T(\rho, \tau|\mathbf{r_0}) \rangle = \sum_n \Delta\langle W_T^{(n)}(\rho, \tau|\mathbf{r_0}) \rangle = \sum_n W_i^{(n)}(\rho|\mathbf{r_0}) \left(\mu_n(\rho_s, \tau) - 2\Im\{\nu_n(\rho_s)\} \right) \Delta\rho_s. \tag{S21}$$

Here, the time harmonic term $e^{-i\omega_i \tau}$ is dropped. Following the marching procedure described in Section S1.1, the temporal correlation function of the total acoustic power after propagating through a 3-D waveguide containing moving random inhomogeneities can be expressed as

$$W_T(\rho, \tau|\mathbf{r_0}) = \sum_n W_i^{(n)}(\rho|\mathbf{r_0}) \exp\left[\int_0^\rho \left(\mu_n(\rho_s, \tau) - 2\Im\{\nu_n(\rho_s\}) \right) d\rho_s \right], \tag{S22}$$

where the modal component of the incident acoustic power, $W_i^{(n)}(\rho|\mathbf{r_0})$, is given in Equation (S18).

The acoustic forward field, $\Phi_T(\mathbf{r}, t|\mathbf{r_0})$, received at $\mathbf{r} = (\boldsymbol{\rho}, z)$ after propagating through a 3-D waveguide containing moving random inhomogeneities can be expressed as

$$\Phi_T(\mathbf{r}, t|\mathbf{r_0}) = e^{-i\omega_i t} \sum_n \chi_T^{(n)}(\boldsymbol{\rho}, t|\mathbf{r_0}) u_n(z), \tag{S23}$$

where $\chi_T^{(n)}(\boldsymbol{\rho}, t|\mathbf{r_0})$ denotes the range- and time-dependent part of each modal component that reflects the stochastic properties of the acoustic field. Second moment of the forward field at a time lag of $\tau = t_1 - t_2$ can then be expressed as

$$\langle \Phi_T(\mathbf{r}, t_1|\mathbf{r_0}) \Phi_T^*(\mathbf{r}, t_2|\mathbf{r_0}) \rangle = e^{-i\omega_i \tau} \sum_n \sum_{n'} \langle \chi_T^{(n)}(\boldsymbol{\rho}, t_1|\mathbf{r_0}) \chi_T^{(n')*}(\boldsymbol{\rho}, t_2|\mathbf{r_0}) \rangle u_n(z) u_{n'}^*(z). \tag{S24}$$

As the acoustic field forward propagates beyond several waveguide depths in range, significant multiple forward scattering effects accumulate and the acoustic modes become statistically uncorrelated. Cross-modal coherence of the range- and time-dependent part of the forward field can then be expressed as

$$\langle \chi_T^{(n)}(\boldsymbol{\rho}, \mathrm{t}_1|\mathbf{r_0})\, \chi_T^{(n')*}(\boldsymbol{\rho}, \mathrm{t}_2|\mathbf{r_0})\rangle = \langle \chi_T^{(n)}(\boldsymbol{\rho}, \mathrm{t}_1|\mathbf{r_0})\rangle\langle \chi_T^{(n')*}(\boldsymbol{\rho}, \mathrm{t}_2|\mathbf{r_0})\rangle + \delta_{nn'}\mathrm{Cov}\left(\chi_T^{(n)}(\boldsymbol{\rho}, \mathrm{t}_1|\mathbf{r_0}), \chi_T^{(n)}(\boldsymbol{\rho}, \mathrm{t}_2|\mathbf{r_0})\right), \quad \text{(S25)}$$

where the double modal sum in Equation (S24) then reduces to a single modal sum as

$$\langle \Phi_T(\mathbf{r}, \mathrm{t}_1|\mathbf{r_0})\, \Phi_T^*(\mathbf{r}, \mathrm{t}_2|\mathbf{r_0})\rangle = e^{-i\omega_i\tau}\,|\langle \Phi_T(\mathbf{r}|\mathbf{r_0})\rangle|^2 + e^{-i\omega_i\tau}\sum_n \mathrm{Cov}\left(\chi_T^{(n)}(\boldsymbol{\rho}, \mathrm{t}_1|\mathbf{r_0}), \chi_T^{(n)*}(\boldsymbol{\rho}, \mathrm{t}_2|\mathbf{r_0})\right)|u_n(z)|^2. \quad \text{(S26)}$$

Analytical expression for the mean total field $\langle \Phi_T(\mathbf{r}|\mathbf{r_0})\rangle$ is given in Equation (S9). To analytically evaluate the second term on the right hand side of Equation (S26), temporal correlation function of the total acoustic power is expressed as

$$\int_0^H dz \frac{1}{d(z)} \langle \Phi_T(\mathbf{r}, \mathrm{t}_1|\mathbf{r_0})\, \Phi_T^*(\mathbf{r}, \mathrm{t}_2|\mathbf{r_0})\rangle = e^{-i\omega_i\tau}\sum_n \langle \chi_T^{(n)}(\boldsymbol{\rho}, \mathrm{t}_1|\mathbf{r_0})\, \chi_T^{(n)*}(\boldsymbol{\rho}, \mathrm{t}_2|\mathbf{r_0})\rangle \quad \text{(S27)}$$

by integrating Equation (S24) through the waveguide depth. Here, modal orthogonality reduces the double modal sum to a single modal sum. Then, by comparing with Equation (S22),

$$\langle \chi_T^{(n)}(\boldsymbol{\rho}, \mathrm{t}_1|\mathbf{r_0})\, \chi_T^{(n)*}(\boldsymbol{\rho}, \mathrm{t}_2|\mathbf{r_0})\rangle = W_i^{(n)}(\rho|\mathbf{r_0})\exp\left[\int_0^\rho (\mu_n(\rho_s, \tau) - 2\Im\{\nu_n(\rho_s\})\, d\rho_s\right]. \quad \text{(S28)}$$

From the definition of the covariance,

$$\mathrm{Cov}\left(\chi_T^{(n)}(\boldsymbol{\rho}, \mathrm{t}_1|\mathbf{r_0}), \chi_T^{(n)}(\boldsymbol{\rho}, \mathrm{t}_2|\mathbf{r_0})\right) = \langle \chi_T^{(n)}(\boldsymbol{\rho}, \mathrm{t}_1|\mathbf{r_0})\, \chi_T^{(n)*}(\boldsymbol{\rho}, \mathrm{t}_1|\mathbf{r_0})\rangle - \langle \chi_T^{(n)}(\boldsymbol{\rho}, \mathrm{t}_1|\mathbf{r_0})\rangle\langle \chi_T^{(n)*}(\boldsymbol{\rho}, \mathrm{t}_2|\mathbf{r_0})\rangle, \quad \text{(S29)}$$

and Equation (S28), Equation (S26) can be analytically evaluated as

$$\begin{aligned}\mathrm{Corr}_{\Phi_T,\Phi_T}(\mathbf{r}, \tau|\mathbf{r_0}) &= \langle \Phi_T(\mathbf{r}, \mathrm{t}_1|\mathbf{r_0})\, \Phi_T^*(\mathbf{r}, \mathrm{t}_2|\mathbf{r_0})\rangle \\ &= |\langle \Phi_T(\mathbf{r}|\mathbf{r_0})\rangle|^2 + \sum_n \left|\Phi_i^{(n)}(\mathbf{r}|\mathbf{r_0})\right|^2 \exp\left[-2\int_0^\rho \Im\{\nu_n(\rho_s)\}d\rho_s\right]\left(\exp\left[\int_0^\rho \mu_n(\rho_s, \tau)d\rho_s\right] - 1\right),\end{aligned} \quad \text{(S30)}$$

where the time harmonic term $e^{-i\omega_i\tau}$ at the acoustic carrier frequency ω_i is dropped. By the definition of covariance, temporal covariance of the acoustic forward field can then be expressed as

$$\begin{aligned}\mathrm{Cov}(\Phi_T(\mathbf{r}, \mathrm{t}_1|\mathbf{r_0}), \Phi_T(\mathbf{r}, \mathrm{t}_2|\mathbf{r_0})) &= \langle \Phi_T(\mathbf{r}, \mathrm{t}_1|\mathbf{r_0})\, \Phi_T^*(\mathbf{r}, \mathrm{t}_2|\mathbf{r_0})\rangle - |\langle \Phi_T(\mathbf{r}|\mathbf{r_0})\rangle|^2 \\ &= \sum_n \left|\Phi_i^{(n)}(\mathbf{r}|\mathbf{r_0})\right|^2 \exp\left[-2\int_0^\rho \Im\{\nu_n(\rho_s)\}d\rho_s\right]\left(\exp\left[\int_0^\rho \mu_n(\rho_s, \tau)d\rho_s\right] - 1\right).\end{aligned} \quad \text{(S31)}$$

As in Equation (S9) for the mean total field, intragration along range in Equation (S31) is numerically evaluated using a finite range slab width, $\Delta\rho_s$, that decouples the distinct modes by satisfying Equation (57) of Reference[1].

S1.3 Modal Field-Temporal-Covariance Coefficient

In this part, the modal field-temporal-covariance coefficient $\mu_n(\rho_s, \tau)$ for two cases are derived. $\mu_n(\rho_s, \tau)$ depends on the relative size of the cross-range coherence length l_y of the random inhomogeneities with respect to the local Fresnel width, $Y_F = \sqrt{\lambda\rho_s(\rho - \rho_s)/\rho}$, where λ is the acoustic wavelength. At slab ranges (ρ_s) close to the source or receiver where cross-range coherence length of the inhomogeneities exceeds the local Fresnel width, the inhomogeneities are fully correlated and 2-D forward scattering process occurs. Within these 2-D scattering regions, analytic expressions for $\mu_n^{2-D}(\rho_s, \tau)$ (Equation (S36)) and $\Im\{\nu_n^{2-D}(\rho_s)\}$ (Equation (S13)) are used to evaluate the integrals in Equation (S31). At slab ranges (ρ_s) further away from

both source and receiver, the local Fresnel width can exceed the cross-range coherence length of the inhomogeneities and result in 3-D scattering[1]. At these 3-D scattering regions, analytic expressions for $\mu_n^{3-D}(\rho_s,\tau)$ (Equation (S35)) and $\Im\{\nu_n^{3-D}(\rho_s)\}$ (Equation (S14)) are used to evaluate the integrals in Equation (S31). At slab ranges where 3-D scattering occurs, first term of the temporal correlation of the scattered field by a single differential range slab of moving inhomogeneities is expressed as

$$
\begin{aligned}
&\langle \Phi_s(\mathbf{r},\mathrm{t}_1|\mathbf{r_0};\Delta\rho_s(\rho_s))\,\Phi_s^*(\mathbf{r},\mathrm{t}_2|\mathbf{r_0};\Delta\rho_s(\rho_s))\rangle_1 \\
&=\sum_m\sum_n\sum_{m'}\sum_{n'} e^{-i\omega_i\tau}\int_0^H dz_t^0\int_0^H dz_{t'}^0 \int_{\rho_s-\frac{\Delta\rho_s}{2}}^{\rho_s+\frac{\Delta\rho_s}{2}} d\rho_t^0 \int_{\phi-\frac{\phi_F}{2}}^{\phi+\frac{\phi_F}{2}} d\phi_t^0\,\rho_t^0\, e^{i\left(\xi_m-\xi_{m'}\right)\left[\rho\rho_t^0/2(\rho-\rho_t^0)\right]\left(\phi_t^0-\phi\right)^2} \\
&\times \frac{4\pi^2 A_c\left(\rho_s,z_t^0,z_{t'}^0\right)}{k(z_t^0)k(z_{t'}^0)d\left(z_t^0\right)d\left(z_{t'}^0\right)}\frac{1}{d^2\left(z_0\right)}\frac{1}{\sqrt{\xi_m\xi_{m'}^*}}\frac{1}{\sqrt{\xi_n\xi_{n'}^*}}\frac{1}{\left(\rho-\rho_t^0\right)\rho_t^0} \\
&\times u_m(z)u_{m'}^*(z)u_n(z_0)u_{n'}^*(z_0)N_m^{(1)}\left(z_t^0\right)N_n^{(1)}\left(z_t^0\right)N_{m'}^{(1)*}\left(z_{t'}^0\right)N_{n'}^{(1)*}\left(z_{t'}^0\right) \\
&\times\ \mathrm{Cov}\Bigg(\left(1+\frac{v_{z_t^0,\mathrm{t}_1}\cos\vartheta_{z_t^0,\mathrm{t}_1}}{C_m^g}\right)s_{z_t^0,\mathrm{t}_1}\left(\pi-\alpha_m(\omega_{nm}),\beta(\phi,\phi_t^0);\alpha_n(\omega_i),\phi_t^0;\omega_{nm}\right)e^{i\Re\{\xi_n-\xi_m\}v_{z_t^0,\mathrm{t}_1}\cos\vartheta_{z_t^0,\mathrm{t}_1}\left(\mathrm{t}_1-\rho/C_m^g\right)}, \\
&\left(1+\frac{v_{z_{t'}^0,\mathrm{t}_2}\cos\vartheta_{z_{t'}^0,\mathrm{t}_2}}{C_{m'}^g}\right)s^*_{z_{t'}^0,\mathrm{t}_2}\left(\pi-\alpha_{m'}(\omega_{n'm'}),\beta(\phi,\phi_{t'}^0);\alpha_{n'}(\omega_i),\phi_{t'}^0;\omega_{n'm'}\right)e^{-i\Re\{\xi_{n'}-\xi_{m'}\}v_{z_{t'}^0,\mathrm{t}_2}\cos\vartheta_{z_{t'}^0,\mathrm{t}_2}\left(\mathrm{t}_2-\rho/C_{m'}^g\right)}\Bigg) \\
&\times e^{i\left(\Re\{\xi_m-\xi_{m'}\}\left(\rho-\rho_t^0\right)+\Re\{\xi_n-\xi_{n'}\}\rho_t^0\right)}e^{-\left(\Im\{\xi_m+\xi_{m'}\}\left(\rho-\rho_t^0\right)+\Im\{\xi_n+\xi_{n'}\}\rho_t^0\right)} \\
&\times e^{i\Re\{\gamma_m+\gamma_n\}z_t^0}e^{-\Im\{\gamma_m+\gamma_n\}z_t^0} \\
&\times e^{-i\Re\left\{\gamma_{m'}+\gamma_{n'}\right\}z_{t'}^0}e^{-\Im\{\gamma_{m'}+\gamma_{n'}\}z_{t'}^0} \\
&+e^{-i\omega_i\tau}\left|\langle\Phi_s(\mathbf{r}|\mathbf{r}_0;\Delta\rho_s(\rho_s))\rangle_1\right|^2, \qquad \text{(S32)}
\end{aligned}
$$

by following the procedure described in Section IV of Reference[1] for the second moment of the scattered field. Equation (S32) is different from equation (76) of Reference[1] because the motion of inhomogeneities is considered and the acoustic time lag is non-zero. In particular, temporal covariance of the product of the inhomogeneities' scatter function density, an amplification factor and a time dependent complex exponential term is required in Equation (S32).

The last term in Equation (S32) is negligibly small by the assumption that the mean scattered field from a differential range slab of inhomogeneities is sufficiently smaller than the mean total field such that the squared mean scattered field is negligibly small. By integrating Equation (S32) over the receiver depth, z, Fresnel width in cross-range, and slab thickness $\Delta\rho_s$, as described in Section IV of Reference[1], the quadruple modal sum reduces to a double modal sum as

$$
\begin{aligned}
&\int_0^H dz\frac{1}{d(z)}\langle \Phi_s(\mathbf{r},\mathrm{t}_1|\mathbf{r_0};\Delta\rho_s(\rho_s))\,\Phi_s^*(\mathbf{r},\mathrm{t}_2|\mathbf{r_0};\Delta\rho_s(\rho_s))\rangle_1 \\
&=\sum_m\sum_n e^{-i\omega_i\tau}\Delta\rho_s\int_0^H dz_t^0\int_0^H dz_{t'}^0\sqrt{\frac{2\pi\left(\rho-\rho_s\right)}{\Re\{\xi_m\}\rho\rho_s}}\frac{4\pi^2A_c\left(\rho_s,z_t^0,z_{t'}^0\right)}{k(z_t^0)k(z_{t'}^0)d\left(z_t^0\right)d\left(z_{t'}^0\right)}\frac{|u_n(z_0)|^2N_m^{(1)}\left(z_t^0\right)N_n^{(1)}\left(z_t^0\right)N_m^{(1)*}\left(z_{t'}^0\right)N_n^{(1)*}\left(z_{t'}^0\right)}{d^2\left(z_0\right)|\xi_m||\xi_n|\left(\rho-\rho_s\right)} \\
&\times\ \mathrm{Cov}\Bigg(\left(1+\frac{v_{z_t^0,\mathrm{t}_1}\cos\vartheta_{z_t^0,\mathrm{t}_1}}{C_m^g}\right)s_{z_t^0,\mathrm{t}_1}\left(\pi-\alpha_m(\omega_{nm}),\phi;\alpha_n(\omega_i),\phi;\omega_{nm}\right)e^{i\Re\{\xi_n-\xi_m\}v_{z_t^0,\mathrm{t}_1}\cos\vartheta_{z_t^0,\mathrm{t}_1}\left(\mathrm{t}_1-\rho/C_m^g\right)}, \\
&\left(1+\frac{v_{z_{t'}^0,\mathrm{t}_2}\cos\vartheta_{z_{t'}^0,\mathrm{t}_2}}{C_m^g}\right)s^*_{z_{t'}^0,\mathrm{t}_2}\left(\pi-\alpha_m(\omega_{nm}),\phi;\alpha_n(\omega_i),\phi;\omega_{nm}\right)e^{-i\Re\{\xi_n-\xi_m\}v_{z_{t'}^0,\mathrm{t}_2}\cos\vartheta_{z_{t'}^0,\mathrm{t}_2}\left(\mathrm{t}_2-\rho/C_m^g\right)}\Bigg) \\
&\times e^{-(2\Im\{\xi_m\}(\rho-\rho_s)+2\Im\{\xi_n\}\rho_s)}e^{i\Re\{\gamma_m+\gamma_n\}z_t^0}e^{-\Im\{\gamma_m+\gamma_n\}z_t^0}e^{-i\Re\{\gamma_m+\gamma_n\}z_{t'}^0}e^{-\Im\{\gamma_m+\gamma_n\}z_{t'}^0}. \qquad \text{(S33)}
\end{aligned}
$$

Furthermore, when $s_{\mathbf{r}_t^0,\mathrm{t}}$ is a zero-mean complex Gaussian random variable and the inhomogeneities horizontal velocity in the forward direction, $v_{z_t^0,\mathrm{t}}\cos\left(\vartheta_{z_t^0,\mathrm{t}}\right)=\bar{u}+u_{z_t^0,\mathrm{t}}$, where $u_{z_t^0,\mathrm{t}}$ is a zero-mean Gaussian random variable, the temporal covariance term in Equation (S33) can be simplified as

$$
\begin{aligned}
&\mathrm{Cov}\Bigg(\left(\left(1+\frac{\bar{u}+u_{z_t^0,\mathrm{t}_1}}{C_m^g}\right)s_{z_t^0,\mathrm{t}_1}(\pi-\alpha_m(\omega_{nm}),\phi;\alpha_n(\omega_i),\phi;\omega_{nm})\,e^{i\Re\{\xi_n-\xi_m\}\left(\bar{u}+u_{z_t^0,\mathrm{t}_1}\right)\left(\mathrm{t}_1-\rho/C_m^g\right)},\right.\\
&\qquad\left.\left(1+\frac{\bar{u}+u_{z_{t'}^0,\mathrm{t}_2}}{C_m^g}\right)s^*_{z_{t'}^0,\mathrm{t}_2}(\pi-\alpha_m(\omega_{nm}),\phi;\alpha_n(\omega_i),\phi;\omega_{nm})\,e^{-i\Re\{\xi_n-\xi_m\}\left(\bar{u}+u_{z_{t'}^0,\mathrm{t}_2}\right)\left(\mathrm{t}_2-\rho/C_m^g\right)}\right)\Bigg)\\
&\approx e^{i\Re\{\xi_n-\xi_m\}\bar{u}\tau}\Bigg[\left(1+\frac{\bar{u}}{C_m^g}\right)^2\langle s_{z_t^0,\mathrm{t}_1}s^*_{z_{t'}^0,\mathrm{t}_2}\rangle\\
&+\frac{1}{\left(C_m^g\right)^2}\left(\langle s_{z_t^0,\mathrm{t}_1}u_{z_t^0,\mathrm{t}_1}\rangle\langle s^*_{z_{t'}^0,\mathrm{t}_2}u_{z_{t'}^0,\mathrm{t}_2}\rangle+\langle s_{z_t^0,\mathrm{t}_1}s^*_{z_{t'}^0,\mathrm{t}_2}\rangle\langle u_{z_t^0,\mathrm{t}_1}u_{z_{t'}^0,\mathrm{t}_2}\rangle+\langle s_{z_t^0,\mathrm{t}_1}u_{z_{t'}^0,\mathrm{t}_2}\rangle\langle s^*_{z_{t'}^0,\mathrm{t}_2}u_{z_t^0,\mathrm{t}_1}\rangle\right)\\
&+\frac{i\Re\{\xi_n-\xi_m\}\tau}{C_m^g}\left(1+\frac{\bar{u}}{C_m^g}\right)\\
&\times\left(2\langle s_{z_t^0,\mathrm{t}_1}u_{z_t^0,\mathrm{t}_1}\rangle\langle s^*_{z_{t'}^0,\mathrm{t}_2}u_{z_t^0,\mathrm{t}_1}\rangle+\langle s_{z_t^0,\mathrm{t}_1}s^*_{z_{t'}^0,\mathrm{t}_2}\rangle\langle u^2_{z_t^0,\mathrm{t}_1}\rangle+\left|\langle s_{z_t^0,\mathrm{t}_1}u_{z_t^0,\mathrm{t}_1}\rangle\right|^2+\langle s_{z_t^0,\mathrm{t}_1}s^*_{z_{t'}^0,\mathrm{t}_2}\rangle\langle u_{z_t^0,\mathrm{t}_1}u_{z_{t'}^0,\mathrm{t}_2}\rangle+\left|\langle s_{z_t^0,\mathrm{t}_1}u_{z_{t'}^0,\mathrm{t}_2}\rangle\right|^2\right)\Bigg]
\end{aligned}
\tag{S34}
$$

by Gaussian moment factorization. The time dependent complex exponential terms in the left hand side of Equation (S34) are linearized before applying Gaussian moment factorization and the scatter function density's argument, $(\pi-\alpha_m(\omega_{nm}),\phi;\alpha_n(\omega_i),\phi;\omega_{nm})$, is ignored for simplicity in the right hand side. When $\bar{u}$ and $u_{z_t^0,\mathrm{t}}$ are comparable and are both smaller than the sound speed, the leading order term in Equation (S34) is $e^{i\Re\{\xi_n-\xi_m\}\bar{u}\tau}\left(1+\bar{u}/C_m^g\right)^2\langle s_{z_t^0,\mathrm{t}_1}s^*_{z_{t'}^0,\mathrm{t}_2}\rangle$ since the other terms are scaled down by square of the modal Mach number. When $\bar{u}=0$, the leading order term reduces to $\langle s_{z_t^0,\mathrm{t}_1}s^*_{z_{t'}^0,\mathrm{t}_2}\rangle$, making the acoustic temporal decorrelation determined by the intrinsic temporal covariance of the inhomogeneities' scatter function density. The higher order terms that include $u_{z_t^0,\mathrm{t}}$ still have relatively negligible effects because they are scaled down by square of the modal Mach number. Keeping the leading order term in Equation (S34) and following the derivation procedures described in Section IV of Reference[1], the modal field-temporal-covariance coefficient can be expressed as

$$
\begin{aligned}
\mu_n^{\text{3-D}}(\rho_s,\tau)=&\sum_m\sqrt{\frac{\rho}{2\pi\Re\{\xi_m\}\rho_s(\rho-\rho_s)}}\frac{1}{|\xi_m|}\left(1+\frac{\bar{u}}{C_m^g}\right)^2\\
&\times\int_0^H dz_t^0\int_0^H dz_{t'}^0 A_c(\rho_s,z_t^0,z_{t'}^0)\frac{4\pi^2}{k(z_t^0)k(z_{t'}^0)d(z_t^0)d(z_{t'}^0)}e^{i\Re\{\xi_n-\xi_m\}\bar{u}\tau}C_{s,s}\left(\rho_s,z_t^0,z_{t'}^0,m,n,\tau\right),
\end{aligned}
\tag{S35}
$$

where $A_c(\rho_s,z_t^0,z_{t'}^0)$ is the coherence area of the inhomogeneities at depths z_t^0 and $z_{t'}^0$. $C_{s,s}\left(\rho_s,z_t^0,z_{t'}^0,m,n,\tau\right)$ quantifies the effects of temporal decorrelation of the inhomogeneities on acoustic temporal decorrelation and expresses coupling between the nth incident and m^{th} outgoing modal plane wave components due to scattering from temporally decorrelating inhomogeneities at depths z_t^0 and $z_{t'}^0$. The definition of $C_{s,s}\left(\rho_s,z_t^0,z_{t'}^0,m,n,\tau\right)$ follows Equation (72) of Reference[1], except the covariance of the scatter function densities, $s_{z_t^0}$ and $s_{z_{t'}^0}$, at zero time lag is replaced by the temporal covariance of the scatter function densities with an acoustic time lag of τ. Equation (S35) is different from Equation (22) of Reference[3] by (1) an amplification factor $\left(1+\bar{u}/C_m^g\right)^2$ and (2) a Doppler spread term, $e^{i\Re\{\xi_n-\xi_m\}\bar{u}\tau}$, both caused by the inhomogeneities' motion. These analytic expressions for $\mu_n(\rho_s,\tau)$ show that acoustic temporal decorrelation can occur by (1) the intrinsic temporal decorrelation of the inhomogeneities through the term $C_{s,s}\left(\rho_s,z_t^0,z_{t'}^0,m,n,\tau\right)$ and (2) Doppler spread caused by the translational motion of the inhomogeneities through $e^{i\Re\{\xi_n-\xi_m\}\bar{u}\tau}$. Effects of both mechanisms accumulate through multiple forward scattering as shown by the integration from source to receiver in Equation (S30). The Doppler spread term leads to spectral broadening of the acoustic temporal correlation, where the spectral width is determined by the incident acoustic frequency, directions of the incident and outgoing modal plane wave components and the modal Mach number of the inhomogeneities' mean forward direction velocity. This frequency spreading can be simply shown by linearizing the Doppler spread term, $e^{i\Re\{\xi_n-\xi_m\}\bar{u}\tau}\approx 1+i\Re\{\xi_n-\xi_m\}\bar{u}\tau$, and substituting the linearized expressions for $\mu_n(\rho_s,\tau)$ into Equation (S30). As an acoustic signal travels to greater ranges and Doppler spread accumulates, this can lead to significant acoustic temporal coherence reduction especially at high frequencies or Mach numbers.

Similarly, the modal field-temporal-covariance coefficient for 2-D scattering regions can be expressed as

$$\mu_n^{\text{2-D}}(\rho_s,\tau) = \sum_m \frac{1}{|\xi_m|}\left(1+\frac{\bar{u}}{C_m^g}\right)^2 \times \int_0^H dz_t^0 \int_0^H dz_{t'}^0 \frac{l_x(\rho_s,z_t^0,z_{t'}^0)}{\xi_m} \frac{4\pi^2}{k(z_t^0)k(z_{t'}^0)d(z_t^0)d(z_{t'}^0)} e^{i\Re\{\xi_n-\xi_m\}\bar{u}\tau} C_{s,s}\left(\rho_s,z_t^0,z_{t'}^0,m,n,\tau\right) \tag{S36}$$

by following an identical procedure as described above. Here $l_x(\rho_s,z_t^0,z_{t'}^0)$ is the coherence length of the inhomogeneities at depths z_t^0 and $z_{t'}^0$. For inhomogeneities with zero forward direction velocity, the Doppler spread term and the amplification factor in Equations (S35) and (S36) vanishes, and so the modal field-temporal-covariance coefficient reduces to Equations (8) and (5) of Reference[4]. Numerical integration over the depth in Equations (S35) and (S36) is conducted using a finite depth increment that is much smaller than the acoustic wavelength.

S2 Statistical Moments of the Scatter Function Density of Surface Waves

Scatter function density per unit area of surface roughness is calculated by the scatter function density of a rough surface subtracted by the scatter function density of a flat surface as shown in Figure S3. This is because interactions with the upper and lower flat boundaries are already included in the Green's function for a waveguide. The mean scatter function density of surface roughness, $\langle s_{\text{roughness}}(\mathbf{k}_s;\mathbf{k}_i)\rangle = 0$ because the mean surface height, $\langle h\rangle = 0$.

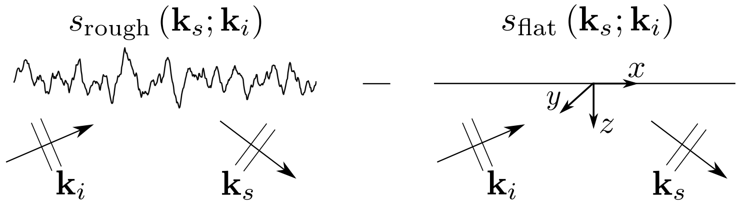

Figure S3. Schematic diagram that shows the scatter function density of surface roughness. Scattered field from surface roughness is the scattered field from a rough surface subtracted by the scattered field from a flat surface. Here $\mathbf{k}_i$ and $\mathbf{k}_s$ are respectively the incident and scattered wavenumber vectors.

The bi-static scattering cross section per unit area of surface roughness can be expressed as

$$\sigma_{\text{SSA}}(\mathbf{k}_s;\mathbf{k}_i) = \frac{\gamma_s^2\gamma_i^2}{(\pi V_z)^2} e^{-(V_z h_{\text{rms}})^2} \iint d\mathbf{r}\, e^{i(\boldsymbol{\xi}_i-\boldsymbol{\xi}_s)\cdot\mathbf{r}} \left(e^{(V_z h_{\text{rms}})^2 R(\mathbf{r})}-1\right), \tag{S37}$$

using small slope approximation of Voronovich[11]. Here, the incident wavenumber vector $\mathbf{k}_i = (\boldsymbol{\xi}_i,\gamma_i)$, where $\boldsymbol{\xi}_i$ is the 2-D incident horizontal wavenumber vector along the XY plane as shown in the axes of Figure S3 and γ_i is the incident vertical wavenumber in the z direction. Similarly, the scattered wavenumber vector $\mathbf{k}_s = (\boldsymbol{\xi}_s,\gamma_s)$. V_z is the z direction component of $\mathbf{V} = \mathbf{k}_i - \mathbf{k}_s$. $R(\mathbf{r})$ is the 2-D spatial correlation coefficient function of a rough surface height and $h_{\text{rms}} = \sqrt{\langle h^2\rangle}$ is the root-mean-square height of the rough surface, where these statistical second moments are determined by the power spectrum of the rough surface, e.g. an isotropic Pierson-Moskowitz sea spectrum. Coherence area of the scatter function density of surface roughness, $A_c(\mathbf{k}_s;\mathbf{k}_i)$, is defined by the effective area that contributes to the magnitude of $\sigma_{\text{SSA}}(\mathbf{k}_s;\mathbf{k}_i)$ as

$$A_c(\mathbf{k}_s;\mathbf{k}_i) = \iint d\mathbf{r} \left|\frac{e^{(V_z h_{\text{rms}})^2 R(\mathbf{r})}-1}{e^{(V_z h_{\text{rms}})^2 R(\mathbf{0})}-1}\right|^2. \tag{S38}$$

When the surface roughness parameter $V_z h_{\text{rms}} \ll 1$, this equation reduces to the $A_c(\mathbf{k}_s;\mathbf{k}_i) = \iint d\mathbf{r}\,|R(\mathbf{r})/R(\mathbf{0})|^2$, which is the coherence area of the rough surface. As the surface roughness parameter, $V_z h_{\text{rms}}$, increases, coherence area of the scatter function density of surface roughness decreases. In a waveguide where multi-modal propagation occurs, both $\sigma_{\text{SSA}}(\mathbf{k}_s;\mathbf{k}_i)$ and $A_c(\mathbf{k}_s;\mathbf{k}_i)$ are functions of incident and scattered acoustic modes and become increasingly incoherent for higher order acoustic

modes because of their high grazing angle. Small slope approximation is adopted here because it inherently satisfies reciprocity and is known to be accurate in the forward direction with low grazing angles[12].

For surface roughness inhomogeneities, expression for the modal field-temporal-covariance coefficient (Equation (S35)) can be simplified as

$$\mu_n^{\text{3-D,Surf}}(\rho_s,\tau) = \sum_m \sqrt{\frac{\rho}{2\pi\Re\{\xi_m\}\rho_s(\rho-\rho_s)}} \frac{1}{|\xi_m|} \left(1+\frac{\bar{u}}{C_m^g}\right)^2 \frac{4\pi^2 A_c(\rho_s,m,n)}{k(0)^2 d(0)^2} e^{i\Re\{\xi_n-\xi_m\}\bar{u}\tau} C_{s,s}(\rho_s,m,n,\tau), \tag{S39}$$

when the surface inhomogeneities are uncorrelated within the local Fresnel width in cross-range direction and 3-D scattering occurs. When surface inhomogeneities, on the other hand, are fully correlated within the cross-range local Fresnel width and 2-D scattering occurs, Equation (S36) simplifies to

$$\mu_n^{\text{2-D,Surf}}(\rho_s,\tau) = \sum_m \frac{1}{|\xi_m|} \left(1+\frac{\bar{u}}{C_m^g}\right)^2 \frac{l_x(\rho_s)}{\xi_m} \frac{4\pi^2}{k(0)^2 d(0)^2} e^{i\Re\{\xi_n-\xi_m\}\bar{u}\tau} C_{s,s}(\rho_s,m,n,\tau), \tag{S40}$$

where $C_{s,s}(\rho_s,m,n,\tau)$ for surface inhomogeneities can be expressed as

$$C_{s,s}(\rho_s,m,n,\tau) = \left|N_m^{(2)}(0)\right|^2 \left|N_n^{(2)}(0)\right|^2 \operatorname{Cov}\left(s_{\rho_s,t_1}(\alpha_m,\phi;\pi-\alpha_n,\phi), s_{\rho_s,t_2}(\alpha_m,\phi;\pi-\alpha_n,\phi)\right). \tag{S41}$$

Here, only the up-going incident and down-going scattered modal plane wave components are retained because these components exclusively interact with the surface inhomogeneities. The covariance of the surface inhomogeneities' scatter function density in Equation (S41) can be expressed in terms of the scattering cross section per unit area as

$$\sigma_{\text{SSA}}(\alpha_m,\phi;\pi-\alpha_n,\phi)\mathscr{R}(\tau) = A_c(\alpha_m,\phi;\pi-\alpha_n,\phi) \frac{\operatorname{Cov}\left(s_{\rho_s,t_1}(\alpha_m,\phi;\pi-\alpha_n,\phi), s_{\rho_s,t_2}(\alpha_m,\phi;\pi-\alpha_n,\phi)\right)}{k(0)^2}, \tag{S42}$$

where $\mathscr{R}(\tau)$ is the temporal correlation coefficient of the rough surface height. This relation between the surface inhomogeneities' scattering cross section per unit area and the covariance of their scatter function density can be derived as follows. The total scattering cross section of a sufficiently large area, A, that exceeds the coherence area of the surface inhomogeneities, A_c, can be expressed as

$$\iint_A d\boldsymbol{\rho}_t \sigma_{\boldsymbol{\rho}_t}(\mathbf{k}_s;\mathbf{k}_i) = \iint_A d\boldsymbol{\rho}_t \iint_A d\boldsymbol{\rho}'_t \left\langle \frac{s_{\boldsymbol{\rho}_t}(\mathbf{k}_s;\mathbf{k}_i)}{k} \frac{s^*_{\boldsymbol{\rho}'_t}(\mathbf{k}_s;\mathbf{k}_i)}{k} \right\rangle, \tag{S43}$$

where $\boldsymbol{\rho}_t$ is the horizontal position of the surface inhomogeneities and $\mathbf{k}_i$ and $\mathbf{k}_s$ are the incident and scattered wavenumber vectors, respectively. When the inhomogeneities are stationary within A and coherent within $A_c(\mathbf{k}_s;\mathbf{k}_i)$,

$$\left\langle \frac{s_{\boldsymbol{\rho}_t}(\mathbf{k}_s;\mathbf{k}_i)}{k} \frac{s^*_{\boldsymbol{\rho}'_t}(\mathbf{k}_s;\mathbf{k}_i)}{k} \right\rangle \approx A_c(\mathbf{k}_s;\mathbf{k}_i) \left\langle \left| \frac{s_{\boldsymbol{\rho}_t}(\mathbf{k}_s;\mathbf{k}_i)}{k} \right|^2 \right\rangle \delta\left(\boldsymbol{\rho}_t - \boldsymbol{\rho}'_t\right), \tag{S44}$$

where $\delta(\boldsymbol{\rho}_t - \boldsymbol{\rho}'_t)$ is a Dirac delta function. Then, the Equation (S43) can be simplified as

$$\iint_A d\boldsymbol{\rho}_t \sigma_{\boldsymbol{\rho}_t}(\mathbf{k}_s;\mathbf{k}_i) = \iint_A d\boldsymbol{\rho}_t A_c(\mathbf{k}_s;\mathbf{k}_i) \left\langle \left| \frac{s_{\boldsymbol{\rho}_t}(\mathbf{k}_s;\mathbf{k}_i)}{k} \right|^2 \right\rangle, \tag{S45}$$

which yields the relation

$$\sigma(\mathbf{k}_s;\mathbf{k}_i) = A_c(\mathbf{k}_s;\mathbf{k}_i) \left\langle \left| \frac{s(\mathbf{k}_s;\mathbf{k}_i)}{k} \right|^2 \right\rangle. \tag{S46}$$

The subscript, $\boldsymbol{\rho}_t$, is dropped since the inhomogeneities are stationary within A. Temporal correlation, or the temporal covariance of the surface inhomogeneities' scatter function density can then be expressed as

$$\sigma(\mathbf{k}_s;\mathbf{k}_i)\mathscr{R}(\tau) = A_c(\mathbf{k}_s;\mathbf{k}_i)\frac{\mathrm{Cov}\left(s_{\mathrm{t}_1}(\mathbf{k}_s;\mathbf{k}_i), s_{\mathrm{t}_2}(\mathbf{k}_s;\mathbf{k}_i)\right)}{k^2}, \tag{S47}$$

where $\mathscr{R}(\tau)$ is the scatter function densities' temporal correlation coefficient function at an acoustic time lag of τ. By using the notations for the modal field-temporal-covariance coefficient, Equation (S47) directly yields Equation (S42).

S3 Statistical Moments of the Scatter Function Density of Bubble Clouds

Mean and second moment of the scatter function density of a random bubble cloud per unit volume are provided using the expressions for discrete inhomogeneities in the Appendix of Reference[1]. Here, randomness of the bubble cloud scatter function density is caused by the random bubble size distribution. Mean scatter function density of a bubble cloud of volume $V_b = A_b D_b$, where A_b and D_b are respectively the horizontal area and penetration depth, can be expressed as[1]

$$\langle s_b \rangle = \frac{\langle S_b \rangle}{V_b} = U n_{\mathrm{v}} \langle S_b \rangle, \tag{S48}$$

where s_b is the scatter function density per unit volume of a random bubble cloud and S_b is the scatter function of a discrete bubble. $n_{\mathrm{v}} = N_b/V_b$ is the number of bubbles per unit volume, where N_b is the total number of bubbles within the bubble cloud. U is a 3-D sinc function defined as

$$U = \frac{1}{V_b}\iiint_{V_b} d\mathbf{u}\, e^{(\mathbf{k}_i - \mathbf{k}_s)\cdot\mathbf{u}}, \tag{S49}$$

where $\mathbf{k}_i$ and $\mathbf{k}_s$ are respectively the incident and scattered wavenumber vectors. The second moment of the scatter function density is[1]

$$\langle |s_b|^2 \rangle = \frac{\langle |S_b|^2 \rangle}{V_b^2} = \frac{1}{V_b}\left[n_{\mathrm{v}}\mathrm{Var}(S_b) + n_{\mathrm{v}}(1 - |U|^2)|\langle S_b \rangle|^2\right] + n_{\mathrm{v}}^2 |U|^2 |\langle S_b \rangle|^2, \tag{S50}$$

where $\mathrm{Var}(S_b)$ is the variance of the scatter function of a single bubble. For bubble clouds of spatial scales much smaller than the acoustic wavelength where $U \approx 1$, scattering from bubbles within the bubble cloud occurs coherently. Equation (S50) then reduces to

$$\langle |s_b|^2 \rangle \approx n_{\mathrm{v}}^2 |\langle S_b \rangle|^2. \tag{S51}$$

At high frequencies where the spatial scales of a bubble cloud exceed the acoustic wavelength, scattering from bubbles within the bubble cloud occurs incoherently. Then Equation (S50) can be approximated as

$$\langle |s_b|^2 \rangle \approx \frac{n_{\mathrm{v}}}{V_b}\langle |S_b|^2 \rangle, \tag{S52}$$

since $U \ll 1$. The mean and second moment of the scatter function density of a bubble cloud are then scaled by the fractional coverage area of bubble clouds to account their patchiness. For example, a typical 1 % fractional areal coverage at 10 m/s wind speed, 25 m^2 horizontal bubble patch area and a penetration depth of 0.5 m are used for calculations in the main text[13].

Scatter function of a single bubble S_b is modeled as a damped-forced oscillator and can be expressed as[14]

$$S_b = \frac{\left(\frac{\omega_0^2}{\omega^2} - 1\right) ka}{\left(\frac{\omega_0^2}{\omega^2} - 1\right)^2 + \delta_{\mathrm{tot}}^2} + i\frac{\delta_{\mathrm{tot}} ka}{\left(\frac{\omega_0^2}{\omega^2} - 1\right)^2 + \delta_{\mathrm{tot}}^2}, \tag{S53}$$

where ω_0 is the Minnaert resonance frequency, ω is the acoustic frequency, k is the acoustic wavenumber and a is the bubble radius. $\delta_{\rm tot}$ is the total damping coefficient including radiation damping and thermal damping given as

$$\delta_{\rm tot} = \delta_{\rm rad} + \delta_{\rm th} = ka + d_{\rm th}\left(\frac{\omega_0}{\omega}\right)^2, \tag{S54}$$

where expression for $d_{\rm th}$ can be found in Reference[15].

S4 Random Realizations of the Acoustic Temporal Coherence from the Power Spectrum of a Measured Acoustic Pressure Field

We describe a procedure that is used to randomly realize the acoustic temporal coherence when the power spectrum of a measured acoustic pressure field is provided. A random acoustic pressure field, $\Phi(t)$ can be realized as

$$\Phi(t) = \sum_{j=1}^{N} A_j \sin\left(\omega_j t + \varepsilon_j\right), \tag{S55}$$

where A_j and ε_j are respectively the amplitude and random phase at frequency ω_j. A_j can be expressed as

$$\frac{1}{2}A_j^2 = S(\omega_j)\Delta\omega, \tag{S56}$$

where $S(\omega_j)$ is the one-sided power spectrum of the acoustic pressure field at frequency ω_j and $\Delta\omega$ is a small but finite frequency difference. This relationship holds since the field variance σ^2

$$\sigma^2 = \int_0^\infty S(\omega)d\omega \approx \sum_{j=1}^{N} S(\omega_j)\Delta\omega = \sum_{j=1}^{N}\frac{A_j^2}{2}, \tag{S57}$$

for a zero-mean stationary random field with independent spectral components. From the power spectrum of a measured acoustic pressure field and a random phase ε_j that follows a uniform distribution between 0 and 2π, the acoustic pressure field can be realized using Equations S55 and S56. Then, for each realization of the acoustic pressure field, the expected acoustic temporal coherence is calculated as

$$\rho(\tau) = \frac{\langle\Phi(t)\Phi(t-\tau)\rangle}{\langle\Phi^2(t)\rangle}, \tag{S58}$$

which are shown as each gray solid lines in Figure 3(b). For this example, the one-sided power spectrum of the measured pressure field is shown in Figure S4.

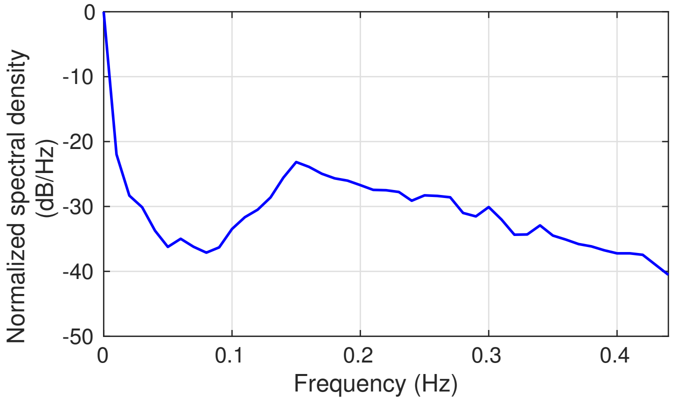

Figure S4. Normalized one-sided power spectrum of a measured acoustic pressure field in October, 1990 in the Barents sea[16].

S5 Doppler Effects on Acoustic Temporal Coherence Reduction as a Function of Frequency

We show the predicted Doppler effects by moving surface gravity waves on acoustic temporal coherence at various frequencies. As shown in Figure S5, Doppler effects are proportional to the acoustic frequency and become notable at high frequencies for fine-scale sensing and communication in the ocean.

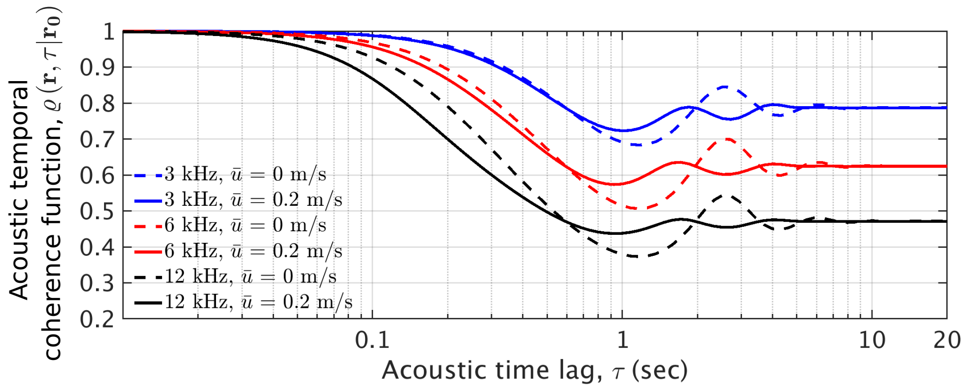

Figure S5. Predicted Doppler effects by moving surface gravity waves on acoustic temporal coherence at various frequencies in the continental-shelf environment in the Gulf of Mexico described in Figure 1(a). Doppler effects are proportional to the acoustic frequency and become notable at high frequencies for fine-scale sensing and communication in the ocean.

S6 Environmental Parameters for Simulations in the Gulf of Mexico, Barents Sea and Bristol Channel

We provide a summary of the required oceanographic parameters that are used to model each environment. For example, the wind speed (or equivalently the significant waveheight) and particle velocity at the sea surface are used as the input to the model for predicting the effects of moving surface gravity waves on acoustic propagation. Similarly, the bubble number densities and internal wave energies are respectively used for modeling the scattering effects of near-sea-surface air bubbles and internal waves.

The acoustic temporal coherence function (Equation (3)) and attenuation (Equation (10)) are calculated using Equation (2), which is expressed in terms of the modal attenuation factor, $\Im\{\nu_n(\rho_s)\}$ (Equations (5) and (6)), and the modal field-temporal-covariance coefficient, $\mu_n(\rho_s, \tau)$ (Equations (7) and (8)). These analytic expressions take the first two statistical moments of the inhomogeneities' scatter function densities as their input. For surface gravity waves, temporal covariance of the scatter function density is calculated using small slope approximation[11] (Equation (S47)), where the required spatial and temporal covariance of the surface waves are calculated using an isotropic Pierson-Moskowitz spectrum[17] at a wind speed given in Table S1. The mean scatter function density of surface gravity waves is zero since the mean surface waveheight is zero. For internal waves,

their scatter function density is modeled using Rayleigh-Born approximation[3,4]. The required spatial and temporal covariance of their waveheights are calculated using a shallow environment Garret-Munk internal wave spectrum[18] at an internal wave energy given in Table S1. The scatter function density of near-sea-surface air bubble clouds is modeled as a damped-forced oscillator, where the measured bubble number density spectrum given in Table S1 is used to calculate the first two statistical moments (Equations (S48) and (S50)).

	Gulf of Mexico	Barents Sea	Bristol Channel
Wind speed (m/s)	5	10	10
Significant waveheight (m)	0.56	2.24	2.24
Surface gravity wave's mean forward-direction particle velocity ($\bar{u}$, m/s)	0.2	0	0
Bubble spectral number density at 100 μm radius (N_0, m^{-3} μm^{-1})	–	2.3×10^8	$2.0 \times 10^7 - 5.0 \times 10^8$
Internal wave energy (J m^{-2})	–	250[19]	–

Table S1. Environmental parameters for simulations in the Gulf of Mexico, Barents Sea and Bristol Channel

S7 Acoustic Coherence Timescales at an Extreme Sea State

We predict the acoustic coherence timescales at an extreme sea state of 20 m/s wind speed in the Barents Sea continental-shelf environment as shown in Figure S6. The e-folding coherence timescale starts to fall below 1 second at 1 km range across the frequencies for wide-area sensing and communication.

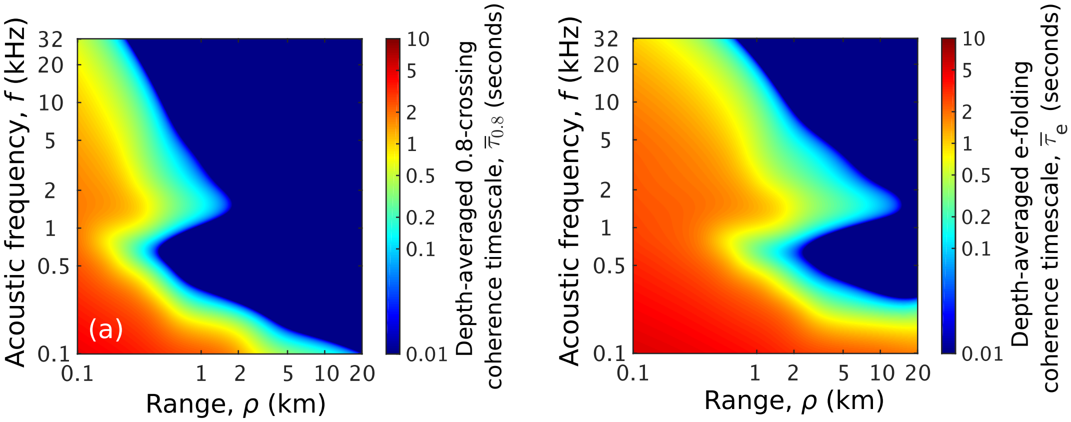

Figure S6. (a) Depth-averaged 0.8-crossing acoustic coherence timescale, $\overline{\tau}_{0.8}$, predicted as a function of frequency and range in the Barents Sea continental-shelf environment (Figure 3(a)) containing surface gravity waves, near-sea-surface air bubbles and internal waves at an extreme sea state of 20 m/s wind speed. 10 % of the sea surface is covered with dense bubbles at such a high sea state[13]. This $\overline{\tau}_{0.8}$ is defined as the timescale over which the acoustic temporal coherence function (normalized acoustic temporal correlation, $\varrho(\mathbf{r}, \tau | \mathbf{r_0})$, defined in Equation (3)) maintains a level above 0.8. (b) Same as (a), but predicting the depth-averaged e-folding acoustic coherence timescale, $\overline{\tau}_e$. The e-folding coherence timescale starts to fall below 1 second at 1 km range across the frequencies for wide-area sensing and communication.

S8 The Effects of Moving Medium on Acoustic Temporal Coherence and Attenuation

In this section, we quantify the effects of the ocean medium's motion on acoustic temporal coherence loss and attenuation. The water particles' slow motion below the sea surface caused by traveling surface gravity waves is considered.

First, we show that the effect of medium motion caused by traveling surface gravity waves on acoustic temporal coherence is negligible compared to that of multiple forward scattering from the rough sea surface in a continental shelf environment. The

total field including multiple forward scattering from the rough sea surface and the medium motion effect can be expressed as

$$\Phi_{\text{Total}}(\mathbf{r},t|\mathbf{r}_0) = \Phi_i(\mathbf{r},t|\mathbf{r}_0) + \Delta\Phi_{\text{surface}}(\mathbf{r},t|\mathbf{r}_0) + \Delta\Phi_{\text{mm}}(\mathbf{r},t|\mathbf{r}_0), \tag{S59}$$

where $\Phi_i(\mathbf{r},t|\mathbf{r}_0)$ is the incident field at a point receiver $\mathbf{r}$ and time t given a point source at $\mathbf{r}_0$, $\Delta\Phi_{\text{surface}}(\mathbf{r},t|\mathbf{r}_0)$ is the scattered field from the traveling rough sea surface that includes 3-D multiple forward scattering effects and $\Delta\Phi_{\text{mm}}(\mathbf{r},t|\mathbf{r}_0)$ is the change in the total field caused by the motion of the medium below the sea surface due to the surface gravity waves. The mean and temporal covariance of $\Phi_i(\mathbf{r},t|\mathbf{r}_0) + \Delta\Phi_{\text{surface}}(\mathbf{r},t|\mathbf{r}_0)$ are given in Equations (S9) and (S31). Change in the total field caused by the medium motion, $\Delta\Phi_{\text{mm}}(\mathbf{r},t|\mathbf{r}_0)$, is calculated using a Parabolic Equation (PE) model[20]. This moving medium effect is calculated by taking the difference between the acoustic fields with a sound speed profile in a motionless medium ($c = c_w(z)$) and a modified sound speed profile due to the water particles' horizontal motion below the sea surface ($c = c_w(z) + u(\rho,z,t)$, where $u(\rho,z,t)$ is the range(ρ)-, depth(z)- and time(t)-dependent random horizontal speed of the water particle). This is possible since an acoustic field propagating through a medium that moves at a low Mach number satisfies the acoustic wave equation in a motionless medium of a sound speed changed by the medium's speed in the acoustic wave propagation direction[21]. A random horizontal water particle speed, $u(\rho,z,t)$, can be realized as

$$u(\rho,z,t) = u_0(z) + \sum_{l=1}^{N} A(\omega_l^{\text{surface}})\omega_l^{\text{surface}} \frac{\cosh\left[k(\omega_l^{\text{surface}})(D-z)\right]}{\sinh\left[k(\omega_l^{\text{surface}})D\right]} \sin\left[k(\omega_l^{\text{surface}})\rho - \omega_l^{\text{surface}} t + \varepsilon_l\right] \Delta\omega^{\text{surface}}, \tag{S60}$$

where $u_0(z)$ and $A(\omega_l^{\text{surface}})$ are respectively the mean Stoke's drift speed and the spectral amplitude of a random 1-D Pierson-Moskowitz sea spectrum at a given wind speed, D is the water depth, $\omega_l^{\text{surface}}$ and $k(\omega_l^{\text{surface}})$ are respectively the angular frequency and wavenumber of the surface gravity waves, $\Delta\omega^{\text{surface}}$ is a small but finite frequency difference and ε_l is a uniformly distributed independent random phase between 0 and 2π. The random horizontal particle speed, $u(\rho,z,t)$, is stationary in range and time with a depth depenedent mean, $u_0(z)$, and standard deviation,$\sigma_u(z)$, where

$$\sigma_u^2(z) = \sum_{l=1}^{N} A(\omega_l^{\text{surface}})\omega_l^{\text{surface}} \frac{\cosh\left[k(\omega_l^{\text{surface}})(D-z)\right]}{\sinh\left[k(\omega_l^{\text{surface}})D\right]} \Delta\omega^{\text{surface}}. \tag{S61}$$

The random $u(\rho,z,t)$ fields at different time instants are correlated and their correlation depends on the time difference, $\tau = |t_2 - t_1|$. For each random realization of $u(\rho,z,t)$, the acoustic fields at time instants $t_1 = 0$ and $t_2 = t_1 + \tau$ are calculated using the PE model. These two acoustic fields are correlated since the time-lagged acoustic field at t_2 is calculated using the sound speed profile modified by the surface gravity waves that progressed over the acoustic time lag, τ, as shown by the time dependence in Equation (S60).

The temporal correlation of the total acoustic field can be expressed as

$$\begin{aligned}
&\langle \Phi_{\text{Total}}(\mathbf{r},t_1|\mathbf{r}_0)\Phi^*_{\text{Total}}(\mathbf{r},t_2|\mathbf{r}_0)\rangle \\
&= \langle (\Phi_i(\mathbf{r},t_1|\mathbf{r}_0) + \Delta\Phi_{\text{surface}}(\mathbf{r},t_1|\mathbf{r}_0) + \Delta\Phi_{\text{mm}}(\mathbf{r},t_1|\mathbf{r}_0))\,(\Phi_i^*(\mathbf{r},t_2|\mathbf{r}_0) + \Delta\Phi^*_{\text{surface}}(\mathbf{r},t_2|\mathbf{r}_0) + \Delta\Phi^*_{\text{mm}}(\mathbf{r},t_1|\mathbf{r}_0))\rangle \\
&= \langle (\Phi_i(\mathbf{r},t_1|\mathbf{r}_0) + \Delta\Phi_{\text{surface}}(\mathbf{r},t_1|\mathbf{r}_0))\,(\Phi_i^*(\mathbf{r},t_2|\mathbf{r}_0) + \Delta\Phi^*_{\text{surface}}(\mathbf{r},t_2|\mathbf{r}_0))\rangle \\
&\quad + \Phi_i(\mathbf{r},t_1|\mathbf{r}_0)\langle \Delta\Phi^*_{\text{mm}}(\mathbf{r},t_2|\mathbf{r}_0)\rangle + \Phi_i^*(\mathbf{r},t_1|\mathbf{r}_0)\langle \Delta\Phi_{\text{mm}}(\mathbf{r},t_2|\mathbf{r}_0)\rangle \\
&\quad + \langle \Delta\Phi_{\text{surface}}(\mathbf{r},t_1|\mathbf{r}_0)\Delta\Phi^*_{\text{mm}}(\mathbf{r},t_2|\mathbf{r}_0)\rangle + \langle \Delta\Phi^*_{\text{surface}}(\mathbf{r},t_1|\mathbf{r}_0)\Delta\Phi_{\text{mm}}(\mathbf{r},t_2|\mathbf{r}_0)\rangle \\
&\quad + \langle \Delta\Phi_{\text{mm}}(\mathbf{r},t_1|\mathbf{r}_0)\Delta\Phi^*_{\text{mm}}(\mathbf{r},t_2|\mathbf{r}_0)\rangle \\
&\approx \langle (\Phi_i(\mathbf{r},t_1|\mathbf{r}_0) + \Delta\Phi_{\text{surface}}(\mathbf{r},t_1|\mathbf{r}_0))\,(\Phi_i^*(\mathbf{r},t_2|\mathbf{r}_0) + \Delta\Phi^*_{\text{surface}}(\mathbf{r},t_2|\mathbf{r}_0))\rangle + \langle \Delta\Phi_{\text{mm}}(\mathbf{r},t_1|\mathbf{r}_0)\Delta\Phi^*_{\text{mm}}(\mathbf{r},t_2|\mathbf{r}_0)\rangle.
\end{aligned} \tag{S62}$$

The last approximate equality holds because (i) $\langle \Delta\Phi_{\text{mm}}(\mathbf{r},t|\mathbf{r}_0)\rangle = 0$ for a slow medium motion as shown in Figure S9 and (ii) $\langle \Delta\Phi_{\text{surface}}(\mathbf{r},t_1|\mathbf{r}_0)\Delta\Phi^*_{\text{mm}}(\mathbf{r},t_2|\mathbf{r}_0)\rangle = \langle \Delta\Phi_{\text{surface}}(\mathbf{r},t|\mathbf{r}_0)\rangle\langle \Delta\Phi^*_{\text{mm}}(\mathbf{r},t|\mathbf{r}_0)\rangle = 0$ given the multiple forward scattered field from the rough sea surface is uncorrelated with the acoustic field change caused by the medium motion and $\langle \Delta\Phi_{\text{surface}}(\mathbf{r},t|\mathbf{r}_0)\rangle = 0$ for a flat mean sea surface. First term in the last relation of Equation (S62) is analytically calculated using Equation (S31), while the last term is numerically estimated from the simulated acoustic field via PE using 100 independent random particle motion realizations (Equation (S60)) at a given wind speed. The effect of the water particles' slow motion below the sea surface is numerically calculated in this way because the change in the field is not caused by multiple forward scattering through the ocean inhomogeneities and the derived analytic expressions do not apply in this case. The acoustic temporal coherence function of the total field is then calculated using Equation (3).

Now we show the effects of multiple forward scattering from traveling surface gravity waves and the motion of the medium on acoustic temporal coherence loss in a continental-shelf environment in Gulf of Mexico with traveling surface gravity waves at a wind speed of 5 m/s (significant waveheight = 0.56 m and WMO sea state 3, Figure 1(a)). A point source is radiating at 17 kHz and a random 1-D Pierson-Moskowitz sea spectrum at this wind speed is used for simulation. Multiple forward scattering from the moving surface gravity waves dominate the acoustic temporal coherence loss (Figures S7(a) and S8(a,d,g)) while the moving medium causes negligible local fluctuations in the temporal coherence including Doppler spread as shown in Figures S7(b-c) and S8(b-c,e-f,h-i).

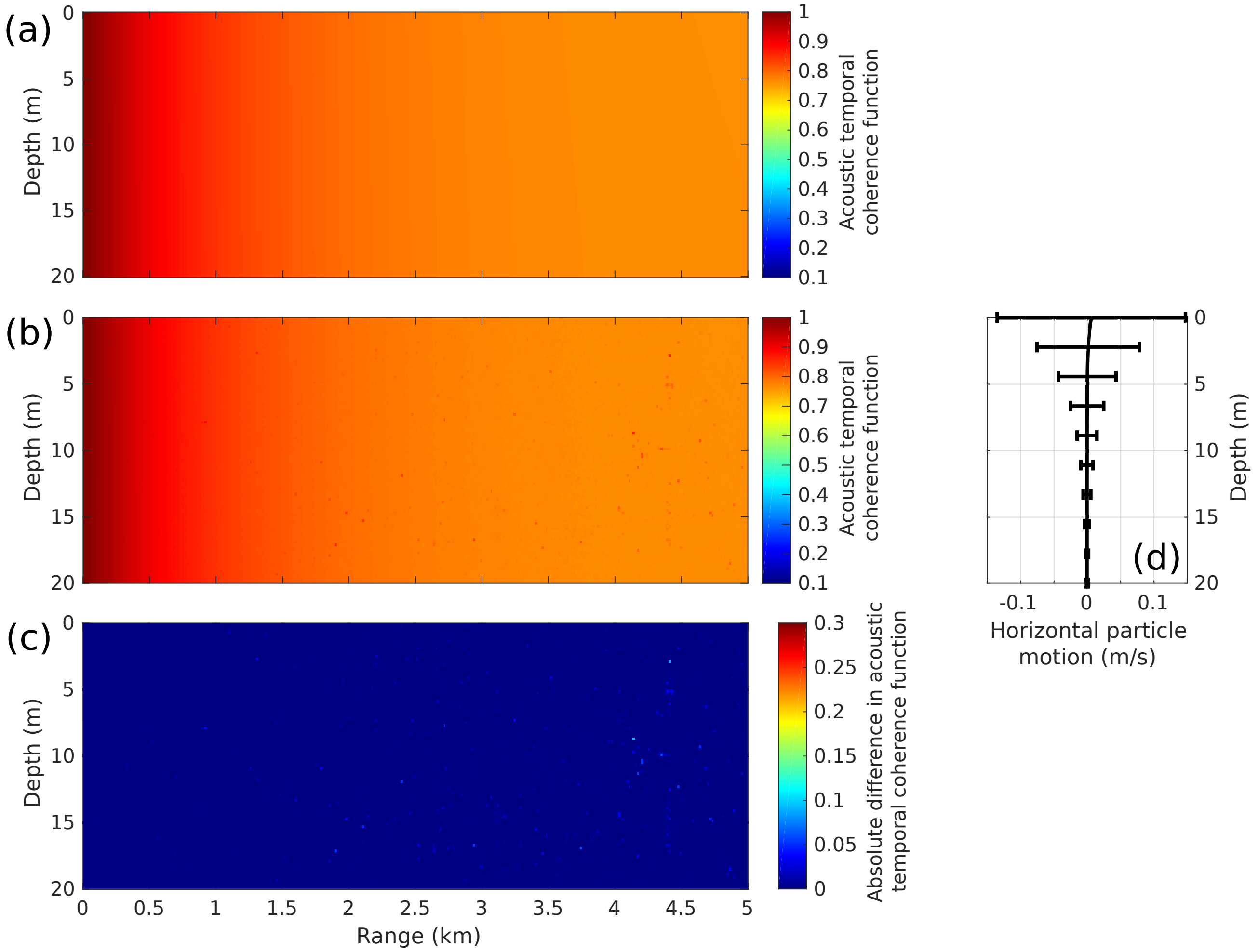

Figure S7. The acoustic temporal coherence function (Equation (3)) at an acoustic time lag of 0.1 seconds in a continental-shelf environment in Gulf of Mexico with traveling surface gravity waves at a wind speed of 5 m/s (significant waveheight = 0.56 m and WMO sea state 3) shown in Figure 1. A point source is radiating at 17 kHz and a random 1-D Pierson-Moskowitz sea spectrum at this wind speed is used for simulation. Multiple forward scattering from the moving surface gravity waves dominate the temporal coherence loss while the moving medium causes negligible local fluctuations in the temporal coherence. (a) Acoustic temporal coherence function including only the multiple forward scattering effect from the traveling surface gravity waves. (b) Same as (a), but including the effects of both multiple forward scattering from the traveling surface gravity waves and the horizontal motion of the medium. (c) Acoustic temporal coherence function change caused by the moving medium (Absolute difference between (a) and (b)). (d) The horizontal particle speed as a function of depth. The solid vertical line shows the mean horizontal Stoke's drift speed and the error bars show standard deviation of the horizontal speed at each depth caused by the particles' orbital motion. The particle motion is generated using the random 1-D Pierson-Moskowitz sea spectrum at this wind speed using Equation (S60).

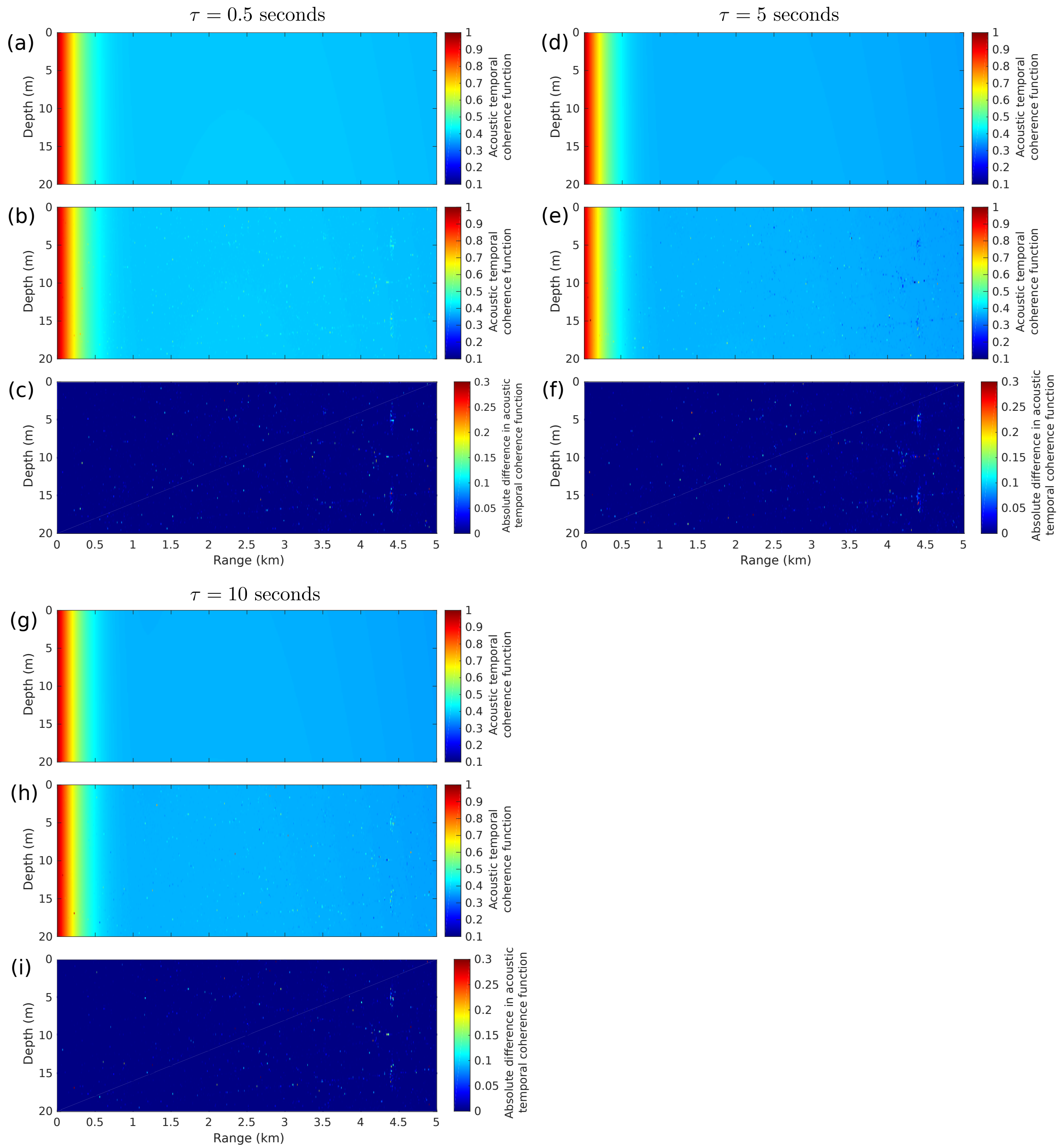

Figure S8. Same as Figure S7, but at different acoustic time lags of $\tau = 0.5, 5$ and 10 seconds. Again, multiple forward scattering from the moving surface gravity waves dominate the temporal coherence loss while the moving medium causes negligible local fluctuations in the temporal coherence. The upper figures (a,d,g) show the acoustic temporal coherence functions including only the multiple forward scattering effects from the surface gravity waves. The middle figures (b,e,h) show the acoustic temporal coherence function including the effects from both multiple forward surface scattering and the medium motion. The lower figures (c,f,i) show the acoustic temporal coherence function change caused by the moving medium (Absolute difference between the upper (a,d,g) and the middle (b,e,h) figures).

We also show that the horizontal motion of the medium has negligible effect on acoustic attenuation in this environment. The medium motion changes the sound speed profile and causes a difference in the Transmission Loss (TL). The change in the acoustic field (Figure S9(c)) due to motion from surface gravity waves or drift in the medium (i) shows no evidence of attenuation with range or depth and (ii) is seen to be negligibly small and fully randomized.

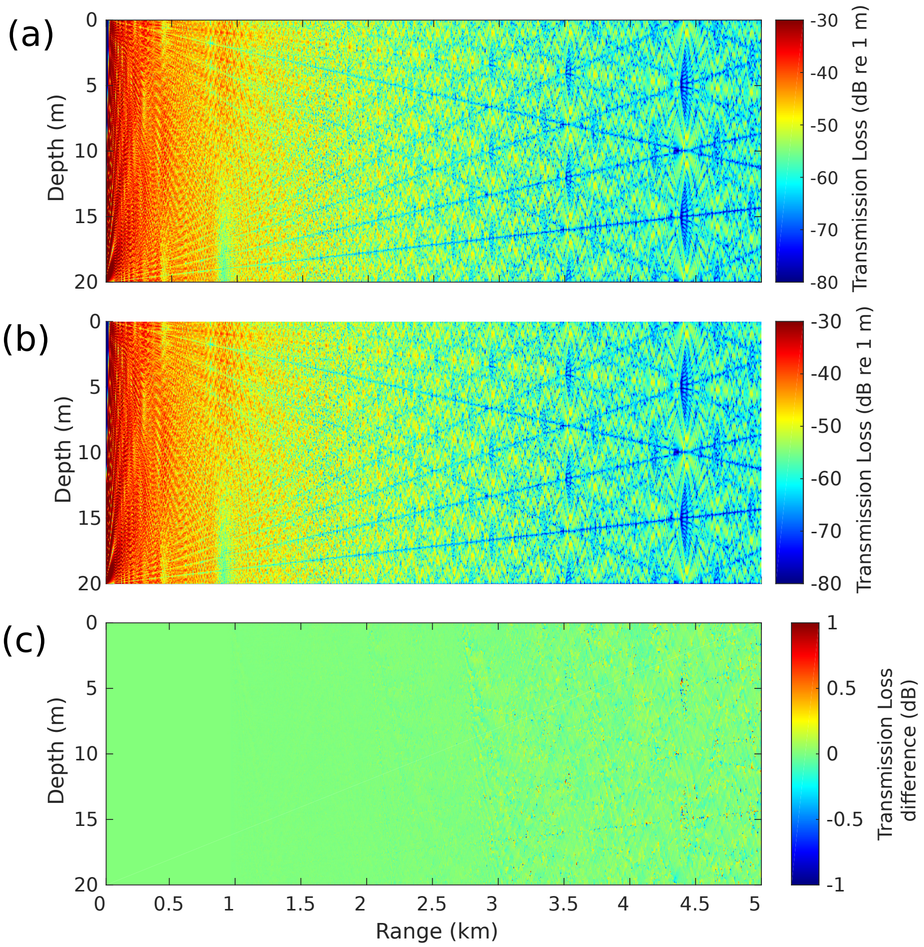

Figure S9. (a) Transmission Loss of the incident field at an arbitrary receiver position, $\mathrm{TL}_i = 10\log_{10}\left(|\Phi_i(\mathbf{r}|\mathbf{r}_0)|^2\right)$, under the continental shelf environment shown in Figure 1(a) at 5 m/s wind speed and 17 kHz source frequency. (b) Same as (a), but the TL including the mean effect of the moving medium, $\mathrm{TL}_{\mathrm{mm}} = 10\log_{10}\left(|\Phi_i(\mathbf{r}|\mathbf{r}_0) + \langle\Delta\Phi_{\mathrm{mm}}(\mathbf{r}|\mathbf{r}_0)\rangle|^2\right)$. The mean acoustic field change caused by the medium motion, $\langle\Delta\Phi_{\mathrm{mm}}(\mathbf{r}|\mathbf{r}_0)\rangle$, is calculated from simulated acoustic fields via a Parabolic Equation (PE) model using 100 independent random water particle motion realizations. The particle motion is generated using the random 1-D Pierson-Moskowitz sea spectrum at this wind speed. (c) Acoustic attenuation (or difference in the TL between (a) and (b)) caused by the horizontal medium motion.

References

1. Ratilal, P. & Makris, N. C. Mean and covariance of the forward field propagated through a stratified ocean waveguide with three-dimensional random inhomogeneities. *J. Acoust. Soc. Am.* **118**, 3532–3559 (2005).

2. Chen, T., Ratilal, P. & Makris, N. C. Mean and variance of the forward field propagated through three-dimensional random internal waves in a continental-shelf waveguide. *J. Acoust. Soc. Am.* **118**, 3560–3574 (2005).

3. Chen, T., Ratilal, P. & Makris, N. C. Temporal coherence after multiple forward scattering through random three-dimensional inhomogeneities in an ocean waveguide. *J. Acoust. Soc. Am.* **124**, 2812–2822 (2008).

4. Gong, Z., Chen, T., Ratilal, P. & Makris, N. C. Temporal coherence of the acoustic field forward propagated through a continental shelf with random internal waves. *J. Acoust. Soc. Am.* **134**, 3476–3485 (2013).

5. Lai, Y.-s. & Makris, N. C. Spectral and modal formulations for the doppler-shifted field scattered by an object moving in a stratified medium. *J. Acoust. Soc. Am.* **113**, 223–244 (2003).

6. Rayleigh, L. Xxxiv. on the transmission of light through an atmosphere containing small particles in suspension, and on the origin of the blue of the sky. *The London, Edinburgh, Dublin Philos. Mag. J. Sci.* **47**, 375–384 (1899).

7. van de Hulst, H. C. *Light scattering by small particles* (Courier Corporation, 1957).

8. Strohbehn, J. W. & Clifford, S. F. vol. 25 of *Topics in applied physics* (Berlin ; New York : Springer-Verlag, 1978.).

9. Ishimaru, A. *Wave propagation and scattering in random media*, vol. 2 (Academic press New York, 1978).

10. Ratilal, P. & Makris, N. C. Extinction theorem for object scattering in a stratified medium. *J. Acoust. Soc. Am.* **110**, 2924–2945 (2001).

11. Voronovich, A. Small-slope approximation in wave scattering by rough surfaces. *Sov. Phys. JETP* **62**, 65–70 (1985).

12. Broschat, S. L. & Thorsos, E. I. An investigation of the small slope approximation for scattering from rough surfaces. part ii. numerical studies. *J. Acoust. Soc. Am.* **101**, 2615–2625 (1997).

13. Monahan, E. C. & Muircheartaigh, I. Optimal power-law description of oceanic whitecap coverage dependence on wind speed. *J. Phys. Oceanogr.* **10**, 2094–2099 (1980).

14. Chen, T. *Mean, variance, and temporal coherence of the 3D acoustic field forward propagated through random inhomogeneities in continental-shelf and deep ocean waveguides*. Ph.D. thesis, Massachusetts Institute of Technology (2009).

15. Eller, A. I. Damping constants of pulsating bubbles. *J. Acoust. Soc. Am.* **47**, 1469–1470 (1970).

16. Sazontov, A. G., Matveyev, A. L. & Vdovicheva, N. K. Acoustic coherence in shallow water: Theory and observation. *IEEE J. Ocean. Eng.* **27**, 653–664 (2002).

17. Pierson, W. J. & Moskowitz, L. A proposed spectral form for fully developed wind seas based on the similarity theory of sa kitaigorodskii. *J. Geophys. Res.* **69**, 5181–5190 (1964).

18. Yang, T. & Yoo, K. Internal wave spectrum in shallow water: Measurement and comparison with the garrett-munk model. *IEEE J. Ocean. Eng.* **24**, 333–345 (1999).

19. Tielbürger, D., Finette, S. & Wolf, S. Acoustic propagation through an internal wave field in a shallow water waveguide. *J. Acoust. Soc. Am.* **101**, 789–808 (1997).

20. Collins, M. D. A split-step padé solution for the parabolic equation method. *The J. Acoust. Soc. Am.* **93**, 1736–1742 (1993).

21. Rayleigh, J. W. S. B. *The theory of sound*, vol. 2 (Macmillan, 1896).
